# Supplementary material for: Supporting Aboriginal Community Controlled Health Services to deliver alcohol care: protocol for a cluster randomised controlled trial
Source: BMJ Open. 2019 Nov 10;9(11):e030909. doi: 10.1136/bmjopen-2019-030909 (PMC6858116; doi:10.1136/bmjopen-2019-030909)
Supplement: Author's manuscript [file bmjopen-2019-030909.draft_revisions.pdf]

BMJ Open

BMJ Open is committed to open peer review. As part of this commitment we make the peer review history of every article we publish publicly available.

When an article is published we post the peer reviewers' comments and the authors' responses online. We also post the versions of the paper that were used during peer review. These are the versions that the peer review comments apply to.

The versions of the paper that follow are the versions that were submitted during the peer review process. They are not the versions of record or the final published versions. They should not be cited or distributed as the published version of this manuscript.

BMJ Open is an open access journal and the full, final, typeset and author-corrected version of record of the manuscript is available on our site with no access controls, subscription charges or pay-per-view fees (http://bmjopen.bmj.com).

If you have any questions on BMJ Open's open peer review process please email info.bmjopen@bmj.com

BMJ Open

Supporting Aboriginal Community Controlled Health Services to deliver alcohol care: protocol for a cluster randomised controlled trial

Journal:	*BMJ Open*
Manuscript ID	bmjopen-2019-030909
Article Type:	Protocol
Date Submitted by the Author:	08-Apr-2019
Complete List of Authors:	Harrison, Kristie; The University of Sydney, Faculty of Medicine and Health<br>Lee, KS; The University of Sydney, Faculty of Medicine and Health; La Trobe University, Centre for Alcohol Policy Research<br>Dobbins, Timothy<br>Wilson, Scott; Aboriginal Drug and Alcohol Council of South Australia, ; The University of Sydney, Faculty of Medicine and Health<br>Hayman, Noel; Inala Indigenous Health Service,<br>Ivers, Rowena; Illawarra Aboriginal Medical Service; The University of Sydney, Faculty of Medicine and Health<br>Haber, Paul; University of Sydney - Camperdown and Darlington Campus, Discipline of Addiction medicine; Sydney Local Health District, Drug Health Services<br>Conigrave, James; The University of Sydney, Faculty of Medicine and Health<br>Johnson, David; Aboriginal Health Council of South Australia<br>Hummerston, Beth; Aboriginal Health Council of South Australia,<br>Gray, Dennis ; Curtin University Bentley Campus, National Drug Research Institute<br>Conigrave, Katherine; University of Sydney, Sydney Medical School
Keywords:	Aboriginal, Torres Strait Islander, alcohol, screening, AUDIT-C, health services

SCHOLARONE™
Manuscripts

Supporting Aboriginal Community Controlled Health Services to deliver alcohol care: protocol for a cluster randomised controlled trial

Kristie H Harrison[1]

KS Kylie Lee[2, 3]

Timothy Dobbins[4]

Scott Wilson[5, 2]

Noel Hayman[6, 7, 8]

Rowena Ivers[9, 1]

Paul S Haber[10, 2]

James H Conigrave[2]

David Johnson[11]

Beth Hummerston[11]

Dennis Gray[12]

Katherine M Conigrave[10, 2]

[1] The University of Sydney, Faculty of Medicine and Health, School of Public Health, NHMRC Centre of Research Excellence in Indigenous Health and Alcohol; C39, University of Sydney, New South Wales 2006 Australia

[2] The University of Sydney, Faculty of Medicine and Health, Discipline of Addiction Medicine, NHMRC Centre of Research Excellence in Indigenous Health and Alcohol; C39, University of Sydney, New South Wales 2006 Australia

[3] La Trobe University, Centre for Alcohol Policy Research, Bundoora, Victoria 3083 Australia

[4] University of New South Wales, National Drug and Alcohol Research Centre, Kensington, New South Wales 2052 Australia

[5] Aboriginal Drug and Alcohol Council Inc.,155 Holbrooks Rd, Underdale, South Australia 5032 Australia

For peer review only - http://bmjopen.bmj.com/site/about/guidelines.xhtml

[6] Southern Queensland Centre of Excellence in Aboriginal and Torres Strait Islander Primary Health Care, PO Box 52, Inala, Queensland 4077 Australia

[7] Griffith University, School of Medicine, Parklands Drive, Southport, Queensland 4215 Australia

[8] University of Queensland, School of Medicine, St Lucia, Queensland 4072 Australia

[9] Illawarra Aboriginal Medical Service, 150 Church St, Wollongong, New South Wales 2500 Australia

[10] Sydney Local Health District, Royal Prince Alfred Hospital, Drug Health Services, Missenden Rd, Camperdown, New South Wales 2050 Australia

[11] Aboriginal Health Council of South Australia, 220 Franklin St, Adelaide, South Australia 5000 Australia

[12] National Drug Research Institute, Curtin University, 7 Parker Place, Technology Park, Bentley Western Australia 6102 Australia

Corresponding author

Kate Conigrave

Drug Health Services, Royal Prince Alfred Hospital

Missenden Road

Camperdown NSW 2050

Phone +61 2 9515 8650 Email: kate.conigrave@sydney.edu.au

For peer review only

ABSTRACT

Introduction

Colonisation and past or current oppression often results in higher prevalence of unhealthy drinking among Indigenous peoples. In Australia, Aboriginal Community Controlled Health Services (ACCHSs) offer culturally accessible care to Aboriginal and Torres Strait Islander (Indigenous) peoples. However there are many competing priorities for staff and clients.

Methods and analysis

A randomised cluster wait-control trial will test the effectiveness of external support in increasing the rate of screening with AUDIT-C and delivery of treatment (brief intervention, counselling or relapse prevention medicines) in ACCHSs across Australia. Twenty-two services will be recruited.

Half the services will receive support soon after the trial commences (intervention or 'early support' services). The wait-control ('late support') services will receive support two years later. Each service will nominate two project champions to advocate for increasing alcohol care. Champions will attend a national training workshop and link together every two months by teleconference to share knowledge. Other support includes onsite training, and second monthly feedback on routinely collected data on screening and treatment provision.

The primary outcome will be rate of screening using AUDIT-C of individuals aged 16+ as routinely recorded on practice software ('Communicare'). Secondary outcomes will be recording of brief intervention, counselling, prescription of relapse prevention medicines; and systolic blood pressure, gamma glutamyltransferase and HbA1c. Multi-level logistic regression will be used to test the effectiveness of support.

Ethics and dissemination

Ethical approval has been obtained from eight ethics committees (Aboriginal-specific where available), including in New South Wales (#1217/16); Central Australia (#CA-17-2842); Northern

For peer review only - http://bmjopen.bmj.com/site/about/guidelines.xhtml

Territory (# 2017-2737); Central Queensland (#17/QCQ/9); Far North Queensland (#17/QCH/45-1143); South Australia (#04-16-694); St Vincents Hospital (Melbourne; #LRR 036/17); and Western Australian (#779). Results will be disseminated through direct reports to ACCHSs, peer-reviewed publications and conference presentations.

Trial registration

Australian New Zealand Clinical Trials Registry (ACTRN12618001892202) retrospectively registered on 21/11/18. Protocol version 1.

For peer review only

Strengths and limitations of this study

- This large cluster randomised controlled trial provides the power to test whether a model of support for Aboriginal and Torres Strait Islander primary care services can increase rates of alcohol screening and treatment provision.
- The protocol has been designed to be compatible with cultural context and to integrate western knowledge with the expertise and holistic approaches of these services.
- The use of regular data feedback and nomination of service champions offers the services an opportunity to be involved in ongoing quality improvement on alcohol care.
- The resultant study will be able to use routinely collected outcome data but this relies on the accurate recording of screening and alcohol care offered to clients.

For peer review only - http://bmjopen.bmj.com/site/about/guidelines.xhtml

INTRODUCTION

Globally, alcohol is the leading cause of death and disease for all people between the ages of 15-49 [1]. Indigenous peoples dealing with colonisation and oppression can be at higher risk of mental illness and harms from alcohol [2]. In Australia, the proportion of Aboriginal and Torres Strait Islander (Indigenous) people who drink alcohol is less than non-Indigenous people [3]. However, Indigenous Australians are 2-8 times more likely to be hospitalised for alcohol-related conditions [4] and nine times more likely to die from alcohol-related harms [5]. This directly impacts on the health gap between Indigenous and non-Indigenous Australians [4]. These increased risks and harms from drinking can be related to experiences of trauma, grief, poverty and cultural dispossession, resulting in increased mental illness, trans-generational trauma and lower life expectancy [2, 4, 6]. The lack of community autonomy and access to culturally appropriate treatment services also contribute [4].

Early detection of unhealthy drinking plays a vital role in reducing the overall burden of alcohol conditions, particularly before people develop health problems or social impacts such as incarceration related to alcohol use [7-9]. Internationally, alcohol screening and brief intervention approaches have been found to be cost-effective in reducing unhealthy drinking, particularly for those who are not dependent on alcohol [10]. Screening for unhealthy drinking aims to detect people who may be drinking above recommended limits [9]. This includes those with hazardous drinking or alcohol use disorders (AUDs) [11]. However across Australian primary care settings, screening and brief intervention for unhealthy drinking is not always standardised [12], informed by Australian National Health and Medical Research Council (NHMRC) drinking guidelines [13], or systematically conducted. It is estimated that 50-70% of people with unhealthy drinking go undetected in Australian primary care [14]. This percentage was the same (50-70% undetected) in four Indigenous primary care settings in Queensland, Australia (in 2001) [15].

Culturally-specific health care services that are led and delivered by Indigenous peoples can have a key role in improving quality of healthcare and treatment access [16]. Aboriginal Community Controlled Health Services (ACCHSs) provide accessible and culturally appropriate health care to

For peer review only - http://bmjopen.bmj.com/site/about/guidelines.xhtml

Indigenous communities across Australia. Each ACCHS has its own holistic-based service delivery model for the communities that they serve [16]. As with mainstream (general population) services, knowledge and practice of screening and brief intervention varies between and within Indigenous primary care services [12]. Some ACCHSs have an alcohol and other drug (AOD) program, which may include an AOD worker, program or team, or links with external AOD specialists/services. Other ACCHSs may have no specific AOD expertise and health practitioners may lack confidence in delivering alcohol care (i.e. screening, brief intervention, counselling or medicines). The uptake of screening and recording of alcohol consumption data varies from service to service [17]. There is an identified need for systems to maintain continuous quality improvement (CQI) in health care delivery, including alcohol treatment, in ACCHSs [18].

There have been minimal studies of culturally-relevant tools or system support models for ACCHSs, with even fewer conducted by Indigenous researchers [4]. AUDIT-C (the Alcohol Use Disorders Identification Test-Consumption) is a 3-item screening tool for alcohol consumption, comprised of the first three questions of the 10-item AUDIT (14). This short form has been shown to have good sensitivity and specificity in comparison to the full AUDIT [19]. Prior studies suggest that AUDIT-C is valid in Indigenous primary care settings, though further study into culturally appropriate delivery is suggested [20-22]. To increase standardisation of alcohol screening in ACCHSs, the use of AUDIT-C has been made a national key performance indicator (nKPI) for ACCHSs nationally [23] and must be reported on using data collected via ACCHS' practice software. This paper outlines the trial protocol for testing if a model of support can assist ACCHSs to integrate the mainstream evidence base on alcohol care with their own cultural and clinical expertise. The use of an inactive control group can be challenging on ethical and partnership grounds, particularly for vulnerable communities. A wait-control design allows support to be delivered to all participating ACCHSs, and enables valid comparisons to be made between intervention and control groups.

For peer review only

For peer review only - http://bmjopen.bmj.com/site/about/guidelines.xhtml

Aims

This trial aims to assess if remote support for ACCHSs can result in increased uptake of evidence-based screening and treatment for unhealthy alcohol use (i.e. drinking above recommended limits). It also aims to develop and evaluate a service-wide model of support for alcohol care in ACCHSs, which builds upon services' existing strengths (see Figure 1).

(Insert Figure 1 about here)

METHODS

Overview

A cluster randomised trial will test the effectiveness of external supports to increase the use of AUDIT-C and evidence-based treatment in ACCHSs. Culturally relevant supports will be offered with the aim of increasing staff skills and capacity to develop, refine and deliver systems for alcohol care. These supports will be based on current evidence and be in-line with services' cultural-care practices. Twenty-two ACCHSs will be recruited across Australia. Services will be randomised into two arms, an early support (intervention) and a late support (wait-control) arm (Figure 2).

(Insert Figure 2 about here)

The support provided will be tailored, guided by ACCHSs, to their individual needs, including needs by remoteness. It will also be underpinned by current evidence, such as national alcohol treatment guidelines [8], Alcohol Treatment Guidelines for Indigenous Australians [24] and national alcohol consumption guidelines [13]. The study methods will be based on principles of conducting health research with Indigenous Australians [25]. This includes quality research, Indigenous involvement and engagement, benefits for Indigenous peoples, and led by Indigenous Australians.

The primary outcome will be routinely collected data on screening rates using AUDIT-C. Secondary outcomes will include recorded delivery of brief intervention, counselling or prescribed medicines to reduce relapse in alcohol dependence. We will also describe changes in reported

For peer review only - http://bmjopen.bmj.com/site/about/guidelines.xhtml

For peer review only

drinking and in biological measures that can be affected by drinking such as gamma glutamyltransferase (GGT; a liver enzyme), HbA1C and systolic blood pressure (BP).

Participating services and eligibility

Recruitment

This trial will recruit whole services (no clients will be recruited). ACCHSs across Australia will be recruited based on expression of interest after circulation of study information to eligible services. A mix of services will be approached (by KH, BH or KC) with a range of remoteness as determined by the Australian Standard Geographical Classification – Remoteness Area (ASGC-RA) [26]. Both verbal and written information will be shared. Face-to-face meetings will be offered to eligible services. Resources will be designated for employing Indigenous project staff in recognition of their specialist skills and knowledge working with ACCHSs. Additional time will be factored into project timelines for engaging with ACCHSs, including liaising with multiple key staff, follow-up conversations, and knowledge translation of research documents.

Inclusion criteria

Services eligible for the trial will meet the following criteria:

1. Are an ACCHS
2. Provide care to 1000+ clients per annum
3. Use Communicare as their practice software

Exclusion criteria

Services will only be excluded if signed institutional consent forms are not returned before the close of recruitment. Data from clients aged 15 or under will not be extracted.

Consent

After written institutional consent is signed, a memorandum of understanding (MOU) will be negotiated by the senior investigator (KC) and lead author (KH) with each ACCHS. Staff participating in individual qualitative interviews will also provide informed consent. Further consent

For peer review only - http://bmjopen.bmj.com/site/about/guidelines.xhtml

will be sought, following the completion of the main trial, if the authors wish to perform ancillary studies using stored data.

Randomisation

ACCHSs will be categorised into three strata based on their remoteness using ASGC-RA [26]: 1) urban and inner regional; 2) outer regional and remote; 3) very remote. Service names will be replaced by consecutive numbers. Within each stratum, half the services will be randomised by a computer program into the early support arm and half into the late support arm. Randomisation will be conducted by an author (TD) who is blind to services' names.

Sample size

Because larger numbers are needed to be able to determine increases in treatment provision than to demonstrate an increase in screening, the treatment provision outcome was used for sample size calculation. In an ACCHS of 1000+ clients per year, approximately 60% are likely to be aged 16 years or older [27]. We estimate that 57% of clients have likely been screened for alcohol use within 12 months [28] and at least 25% of clients who were screened are likely to be drinking above NHMRC recommended limits [13, 29, 30]. In the late support arm, it is likely that 60% of identified unhealthy drinkers will have an alcohol intervention recorded [28]. Assuming an Intra-cluster correlation coefficient (ICC) of 0.04 [31, 32], enrolling 10 early support services and 10 late support services will allow for an increase in treatment provision of at least 13% in the early support services to be detected (i.e. from 60% to 73%; 80% power and 2-sided significance of 0.05). We will enrol an additional service in each arm to allow for the possibility of clusters dropping out of the study. Accordingly, the target number of services to be enrolled is 22.

The model of support

ACCHSs will be acknowledged as the experts in cultural health care for Indigenous peoples in their communities and Australia. The study also recognises the need for flexibility based on needs of individual services and of the ACCHS sector as a specialist sector overall. Support approaches will

For peer review only - http://bmjopen.bmj.com/site/about/guidelines.xhtml

include the same key elements (Table 1). However approaches will be tailored to the needs of each ACCHS, for what works best for their service, clients and communities.

Table 1. Key support elements

**1.**	**Service champions**	Services asked to nominate two representatives to act as advocates of alcohol care and links between the service and research team
**2.**	**National workshop**	National capacity building workshop for service champions at the start of the support phase (and for early support services, a wrap-up feedback workshop at the end of the maintenance support phase)
**3.**	**Onsite training**	Training will be offered to each ACCHS at their service
**4.**	**Resources/funding**	Some resources will be given to services for free with additional funding provided for the selection and purchase of further resources.
**5.**	**Practice software (Communicare) support**	Support will be provided to services throughout the trial to facilitate the routine clinical use of alcohol-related items in the practice software.
**6.**	**Data feedback**	Individual service data will be fed back to each service every second month
**7.**	**Phone conferences**	Phone conferences will be held every second month between service champions and project team (including an addiction medicine physician). These will allow sharing of ideas and joint problem solving.
**8.**	**Online platform**	Further resources and information will be shared via a secure online platform.

For peer review only - http://bmjopen.bmj.com/site/about/guidelines.xhtml

Continuous refinement of support approaches will occur during the trial, informed by qualitative and quantitative data and service feedback. Early support ('intervention') services will receive the support soon after the trial starts. Late support (wait-control) services will receive the same intervention elements two years later. Due to the nature of the intervention, it is not possible to blind participants or staff as to which services are receiving active support.

1. Identifying service champions

Each service will be asked to identify two representatives to work collaboratively with their service staff and the research team on increasing alcohol care. Services will be asked to consider nominating a clinician, Aboriginal health professional and/or an individual from management. Service champions will be invited to attend an initial national face-to-face workshop, and second-monthly teleconferences. These meetings will ensure that support approaches are in line with the values of each ACCHS, and will allow sharing of expertise and initiatives. Champions will be key in highlighting the potential benefits of change in their service and will encourage service staff to discuss their second monthly data feedback, and to address any key barriers to change [33].

2. Workshops

A national workshop will be held at the beginning of each support phase to bring champions from those services together for capacity building and networking. Feedback will be obtained from champions about pre- and post-workshop knowledge, training preferences, alcohol priority areas, and useful resources. Key presentations from workshops will be video-recorded and made available via a password-protected website.

A wrap-up workshop will also be organised for early support service champions (only) at the end of their support phase. Opportunities will be provided for service champions to provide verbal and written feedback and for CQI and networking. Study results to that point will be discussed.

For peer review only

3. Onsite training

Training will be offered at each service for all staff, with maximum duration of two days. Core elements will include screening, alcohol and the body, brief intervention and treatment approaches, including relapse prevention medicines. Due to the individuality of each service, flexibility will be given to allow inclusion of other (alcohol-related) training topics. The training will be co-facilitated by an Addiction Specialist (KC) and an Aboriginal researcher with clinical background (KH) and will use culturally specific content and resources. Training content will be aligned with Indigenous protocols such as gender appropriateness, kinship systems and cultural obligations; such a family and community sharing, which at times can impact on substance use [21, 34]. Pre- and post-training feedback will be collected from participants.

4. Resources / Funding

The trial will provide several hard copy and electronic resources for free to services (i.e. the Handbook for Aboriginal Alcohol and Drug Work [35], Alcohol Awareness Kit [35] and Quick Reference Guide to the Treatment of Alcohol Problems [36]).

Each ACCHS will be provided with funding to purchase additional resources. Services can choose, in partnership with the chief investigators, how they spend their funding. The early support arm of the project will receive up to $9,000 for resources. The late support arm will receive up to $3,500. This is a reduced sum as they will have access to resources developed within the early support phase, so time and development costs will be less. Resources must be alcohol-related and may include additional training, conference attendance, resources for staff or clients, funds for health promotion events or for local adaptation of resources (e.g. translation into local language/s).

5. Practice software support

A Communicare officer will be available during the trial to support ACCHSs with the practice software. Some ACCHSs may choose to make modifications to Communicare to make alcohol 'clinical items' more accessible for staff. One example is adding AUDIT-C into the Aboriginal and

For peer review only - http://bmjopen.bmj.com/site/about/guidelines.xhtml

Torres Strait Islander (Indigenous) health assessment ('Adult Health Check'), which was not routine in standard versions of Communicare at the time of trial commencement. AUDIT-C can also be added to other regular assessments such as: antenatal and the pre-consult examination (i.e. routine observations and/screening by a nurse or Aboriginal health worker before seeing the doctor). Services will also be provided with information about existing 'clinical items' available on Communicare for recording data for episodes of alcohol care (education/advice; counselling), which can then be retrieved by data extraction.

6. Second monthly data feedback

Individual services will receive feedback on their routinely collected data on alcohol care every two months during their support phase. Data will be represented visually using images and or graphs (Figure 3) and be fed back to nominated staff, including champions. Services will be asked for feedback on the data representation and adaptions will be made as requested. Services will be encouraged to discuss the results among staff, to see if there are ways to further improve alcohol screening and treatment rates.

(Insert Figure 3 about here)

7. Phone conferences

Second-monthly phone conferences will be organised for service champions. These conferences will provide the opportunity for ACCHSs to network nationally. The champions will be split into two groups for the phone conferences, based on remoteness. During teleconferences, champions can discuss their data feedback, progress, successes, difficulties, helpful tools, resources or approaches.

8. Online platform

A password-protected web portal will allow ACCHS staff to access and share current evidence, culturally appropriate resources, and to network. It will include a discussion board. Short videos, training presentation slides, and clinical protocols will be uploaded by project staff.

For peer review only

Data collection

Qualitative data

Qualitative data will be collected from ACCHSs at the start of the early support arm. The data will help inform the support model. Structured phone interviews will be conducted with approximately five staff members from each ACCHS in the early support arm, with the aim of a total of fifty. Champions and/or key staff will be approached to assist with recruiting interviewees.

Alcohol can be sensitive to discuss due to cultural protocols such as kinship/family systems, gender or age, shame (embarrassment) or community and social acceptance of alcohol [37-39]. Therefore, the interview questions will be framed using a strengths-based, capacity-building approach [40]. Interview questions will include: approaches to make it easier to talk about alcohol, treatment around alcohol, skills and knowledge needed to provide quality alcohol care, new ways of helping people who struggle with alcohol, other successful programs that could be used as a model for alcohol care, and ideas for improvement in alcohol care.

Interviews will be conducted by two Indigenous staff with experience working with ACCHSs and conducting interviews. One interviewer will facilitate the interview while the other will live-transcribe. The interviewers will compare and discuss notes after each interview. Memos will also be written by the lead author (KH) on each interview.

Quantitative data

Non-identifiable data on episodes of screening and alcohol care and related variables (see below) will be extracted from the Communicare database of each ACCHS on the 28th day of every second month throughout the 5-year trial (Figure 4). The baseline extraction will include data for 12 months before the trial starts. Participating services use a Structured Query Language (SQL) report developed for this project to extract data, then will send data to the project team. Data will include AUDIT-C responses, and the client's drinking status as perceived by a health professional ('Non-drinker', 'Within safe drinking limits' or 'Unsafe - needs intervention'); record of alcohol brief

For peer review only - http://bmjopen.bmj.com/site/about/guidelines.xhtml

intervention, counselling, and medicines prescribed for relapse prevention (i.e. acamprosate, naltrexone or disulfiram).

(Insert Figure 4 about here)

Other data extracted will include age, gender, last date the client was seen, and biological markers which may be affected by alcohol consumption (BP, HbA1C and GGT).

A spreadsheet will track dates when key elements of support are provided to each service.

Service retention and withdrawal

Regular contact will be made with services during their support phase through second monthly teleconferences (by KC and often KH), and also for the purposes of data extraction (by BH). Every effort will be made to address any concerns or suggestions, and to minimise any burden of participation on the services. However, services may withdraw from the study, for any reason and at any time.

Data storage and confidentiality

Data, and study-related material will be stored securely in a University of Sydney drive only accessible by the research team. To protect confidentiality, identification numbers rather than names are used for services in the data set and reporting. Client names are never provided to the research team. Investigators involved in data analysis, and selected administrative staff will be granted access to study data.

For peer review only - http://bmjopen.bmj.com/site/about/guidelines.xhtml

For peer review only

Analysis

Qualitative

Two independent analyses of the qualitative data will be conducted: analysis of the memos and of the transcripts. This complementary analytical strategy aims to enhance examination of the raw data in the formation of key concepts [41].

Interview transcripts and memos will each be imported into the qualitative software, NVivo (Version 11). Memos will be analysed by the lead interviewer (KH). Transcripts from the structured interviews will be analysed by an independent research associate to produce key themes. This involves the interpretation of descriptive data to explain the meanings of the interviewees' responses [42]. Each data set will be coded separately using a process of constant comparison [42]. This will involve coding and organising likened data into categories along with a comparison and interpretation of emerging themes across all transcripts and memos [43]. A team of relevant experts will meet to discuss both analyses and find consensus on key themes and sub-themes. These key themes and findings will be described.

Quantitative

Only the data on Indigenous Australian clients age 16+ years who have attended a participating ACCHS in the past 12 months will be included in analysis to assess:

1. The number of clients who are screened using AUDIT-C
2. The number of clients identified with unhealthy alcohol use
3. The number of clients who are offered treatment, including advice/education or counselling, relapse prevention medicines

We will calculate AUDIT-C screening rates before and after support is provided; and records of treatment provided. These data will be compared between the two study arms.

Characteristics of ACCHSs, such as number of clients, will be compared descriptively between the early- and late-support services. Characteristics that display major imbalance between arms will be considered as adjustment factors in outcome analyses.

For peer review only - http://bmjopen.bmj.com/site/about/guidelines.xhtml

For peer review only

Multi-level logistic regression will then be performrmed to assess whether rates of screening and treatment provision differ between early- and late-support services. The multi-level logistic regression model accounts for episodes of screening being nested within clients, who are themselves nested within the services they attend. We will perform unadjusted analyses, as well as adjusting for characteristics that are unbalanced between study arms. We will use statistical packages, SPSS for descriptive analyses and R for multi-level modelling. As data is collected as part of routine clinical activity, missing data is likely to be non-random, as such multiple-imputation will not be performed.

We will assess if clients are more likely to be screened and offered treatment during periods when their service is being supported. A secondary analysis using multi-level regressions, will assess if the support offered is associated with changes in the recorded prevalence of unhealthy drinking (using AUDIT-C) or changes in biological markers that can be affected by alcohol (GGT, HbA1c and BP).

Ethical approvals

Ethical approval has been obtained from eight ethics committees: The Aboriginal Health and Medical Research Council of New South Wales (1217/16); Central Australian Human Research Ethics Committee (CA-17-2842); Northern Territory Department of Health and Menzies School of Health Research (2017-2737); Central Queensland Hospital and Health Service (17/QCQ/9); Far North Queensland (17/QCH/45-1143); Aboriginal Health Research Ethics Committee, South Australia (04-16-694); St Vincent's Hospital (Melbourne) Human Research Ethics Committee (LRR 036/17); and Western Australian Aboriginal Health Ethics Committee (779).

As there are minimal foreseeable risks in this study, there will not be an independent committee monitoring conduct of the trial, data or adverse events. Feedback from services will be provided to the investigators and adaptations made as necessary.

For peer review only - http://bmjopen.bmj.com/site/about/guidelines.xhtml

For peer review only

Patient and public involvement in the research

This trial was suggested by the Aboriginal Health Council SA, the umbrella organisation for South Australian community controlled health services. Service participation in the trial must be approved by the Board of each community-controlled health service. The Board is recognised as representing patients and community in such decisions. As described above, service staff will be consulted throughout their support phase on how the project can best support them. Results will be fed back bimonthly to services in an accessible manner for all staff and Board members, and services will have opportunity to provide comment on draft outcome reports before publication.

Dissemination of findings

As described above, results will be reported directly to participating ACCHSs. Results will then be disseminated via peer-reviewed publications and conference presentations. Publications will be led by study investigators, research students or staff supported by the investigators. The resources developed through this trial will also be made freely available electronically at the end of the study period to support other health services.

Data sharing

It is not possible to make the dataset publicly available because of ethical constraints. Alcohol is a sensitive issue, and the data belongs jointly to the 22 services who take part. For the main outcome report, statistical code used to analyse data will be made publicly available.

Authorship policy

All grant holders, and those involved in study conception and design, will co-author the main outcome piece. Further articles may be produced based on sub-analyses, or on ancillary research questions. Those papers will be authored by those directly involved in those research questions. Acknowledgement will be given to individuals involved in the design of the main paper.

For peer review only

For peer review only - http://bmjopen.bmj.com/site/about/guidelines.xhtml

Protocol amendments

The trial's registration data is shown at Table 2 (ACTRN12618001892202). If any protocol amendments are necessary, these be agreed on by study authors, and where appropriate, participating services. Then they will be registered with the Australian and New Zealand Clinical Trials Registry.

Table 2. WHO trial registration data

Data category	Information
Primary registry and trial identification number	Australia New Zealand Clinical Trials Registry: ACTRN12618001892202
Date of registration in primary registry	2018-11-21
Secondary identifying numbers	APP1105339
Sources of monetary or material support	National Health and Medical Research Council (NHMRC; Australia)
Primary sponsor	The University of Sydney
Secondary sponsor	Royal Prince Alfred Hospital
Contact for public and scientific queries	Kate Conigrave, MBBS, FAChAM, FAFPHM, PhD<br>kate.conigrave@sydney.edu.au
Public title	Supporting Aboriginal Community Controlled Health Services to deliver alcohol care: a cluster randomised controlled trial
Scientific title	Increasing uptake of evidence-based management of unhealthy alcohol use in Aboriginal primary health care services: a cluster randomised controlled trial
Counties of recruitment	Australia
Health condition(s) or problem(s) studied	Hazardous alcohol use; Alcohol use disorders

For peer review only - http://bmjopen.bmj.com/site/about/guidelines.xhtml

Data category	Information
Interventions	Health service support (including training, sharing learning between services); regular data feedback
Key inclusion or exclusion criteria	Health services:<br>1. are Aboriginal Community Controlled Health services<br>2. deliver care to at least 1000 unique clients annually<br>3. use 'Communicare' as their practice software<br>Data from clients 16 years and older are eligible for extraction
Study type	Interventional;<br>Allocation: randomised;<br>Primary purpose: prevention;<br>Cluster randomised trial
Date of first enrolment	28/10/2016
Target sample size	22 services
Recruitment status	Complete
Primary outcomes	AUDIT-C screening rate
Key secondary outcomes	Brief intervention for alcohol rate

For peer review only - http://bmjopen.bmj.com/site/about/guidelines.xhtml

DISCUSSION

To our knowledge, this is the first large-scale randomised trial testing whether external support can enhance uptake of evidence-based alcohol care in services for Indigenous peoples. This study will add knowledge in the field of screening, early intervention and treatment for Indigenous peoples of Australia and inform future research and policy development. The findings are also likely to be relevant for Indigenous peoples internationally who have similar experiences of colonisation and inter-generational trauma. The research will also have relevance for non-Indigenous health services.

The model of support is designed to be culturally appropriate and collaborative, and tailored to individual service needs. The model is based on the use of champions within services, and feedback of routinely collected data, and so has potential to be sustainable, allowing for continuing quality improvement. The model also has potential to be scaled up for longer-term support of ACCHSs Australia-wide.

Acknowledgements

This work was supported by the NHMRC funding through a Project Grant (#1105339); a Centre of Research Excellence in Indigenous Health and Alcohol (#1117198); and a Practitioner Fellowship for K. Conigrave (#1117582). The funding body had no contribution to study design, writing of report, or in the decision to submit this report for publication; nor will they influence the submission of future reports. Thanks to Dr David Scrimgeour and to the Aboriginal Health Council of South Australia for suggesting this study. Also to Professor Sandra Eades, Professor Robert Sanson-Fisher and Mr Paul Ishiguchi for advice on study design and management.

Authors' contributions

KH refined the protocol for cultural appropriateness, drafted the protocol report; reviewed literature, recruited services, and will play a key role in training, qualitative data collection and qualititative analysis. KC, PH, NH, RI, KL conceived the study design. SW, NH assisted with ensuring design was culturally appropriate; KC,PH,RI, provide clinical expertise; BH and DJ provided guidance on

For peer review only - http://bmjopen.bmj.com/site/about/guidelines.xhtml

Communicare use and data extraction and ACCHS support; BH assisted with service recruitment leads data extraction, and facilitates service liaison. TD developed the analysis plan, including power analyses, and provides ongoing advice. JC is responsible for merging and curating the data, and conducting t_1 quantitative analysis. KC, PH, RI, DG, TD, KL, SW, DJ are grant holders. All grant holders are on the project steering committee; all authors contributed to the development of the study protocol and approved this manuscript.

Declaration of interests

The principle investigators have no financial or other competing interests.

Trial Sponsor

The University of Sydney, NSW 2006, Australia and Sydney Local Health District

Word Count: 3396 (excluding tables, and acknowledgements onwards)

For peer review only

For peer review only - http://bmjopen.bmj.com/site/about/guidelines.xhtml

References

1. GBD 2016 Risk Factors Collaborators, *Global, regional, and national comparative risk assessment of 84 behavioural, environmental and occupational, and metabolic risks or clusters of risks, 1990–2016: a systematic analysis for the Global Burden of Disease Study 2016.* Global Health Metrics, 2016. **Vol 390 September 16, 2017**.
2. Kirmayer, L.J., G.M. Brass, and C.L. Tait, *The mental health of Aboriginal peoples: transformations of identity and community.* Canadian Journal of Psychiatry, 2000. **45**(7): p. 607-16.
3. Australian Bureau of Statistics/Australian Institute of Health and Welfare, *The health and welfare of Australia's Aboriginal and Torres Strait Islander peoples*. 1999, ABS: Canberra.
4. Australian Institute of Health and Welfare, *Substance use among Aboriginal and Torres Strait Islander people*. 2011, Canberra: Australian Institute of Health and Welfare.
5. Australian Institute of Health and Welfare, *Aboriginal and Torres Strait Islander health performance framework, 2008 report: detailed analyses*. Cat. no. IHW 22. 2008, Canberra: AIHW.
6. Eckermann, A.-K., T. Dowd, and E. Chong, *Binan Goonj: bridging cultures in Aboriginal health*. 2010: Elsevier Australia.
7. Kaner, E.F.S., et al., *The effectiveness of brief alcohol interventions in primary care settings: a systematic review.* Drug and Alcohol Review, 2009. **28**(3): p. 301-323.
8. Haber, P., et al., *Guidelines for the treatment of alcohol problems*. 2009, Canberra: Australian Government Department of Health and Ageing.
9. Chapman, C., et al., *Delay to first treatment contact for alcohol use disorder.* Drug and alcohol dependence, 2015. **147**: p. 116-121.
10. Solberg, L.I., M.V. Maciosek, and N.M. Edwards, *Primary care intervention to reduce alcohol misuse: ranking its health impact and cost effectiveness.* American Journal of Preventive Medicine, 2008. **34**(2): p. 143-152.
11. World Health Organisation, *ICD-11 for mortality and morbidity statistics (ICD-11 MMS)* June 18 2018 ed. 2018: World Health Organisation.
12. Clifford, A., A. Shakeshaft, and C. Deans, *How and when health-care practitioners in Aboriginal Community Controlled Health Services deliver alcohol screening and brief intervention, and why they don't: A qualitative study.* Drug and Alcohol Review, 2012. **31**(1): p. 13-19.
13. National Health and Medical Research Council, *Australian guidelines to reduce health risks from drinking alcohol*. 2009, Canberra, Australia: Commonwealth of Australia.
14. Reid, A.L.A., et al., *General practitioners' detection of patients with high alcohol intake.* British Medical Journal, 1986. **293**: p. 735-737.
15. d'Abbs, P., et al., *The Grog Mob: lessons from an evaluation of a multi-disciplinary alcohol intervention for Aboriginal clients.* Australian and New Zealand Journal of Public Health, 2013. **37**(5): p. 450-456.
16. Campbell, M., J. Hunt, and AHMRC, *Aboriginal communities improving Aboriginal health: An evidence review on the contribution of Aboriginal Community controlled Health Services to improving Aboriginal health*. 2015, Sydney: Aboriginal Health and Medical Research Council, NSW.
17. Clifford, A., A. Shakeshaft, and C. Deans, *Training and tailored outreach support to improve alcohol screening and brief intervention in Aboriginal Community Controlled Health Services.* Drug and Alcohol Review, 2013. **32**(1): p. 72-79.
18. Australian Institute of Health and Welfare, *National key performance indicators for Aboriginal and Torres Strait Islander primary health care First national results June 2012 to June 2013.* 2018.
19. Bradley, K., et al., *AUDIT-C as a brief screen for alcohol misuse in primary care.* Alcoholism: Clinical and Experimental Research, 2007. **31**: p. 1208-17.

For peer review only - http://bmjopen.bmj.com/site/about/guidelines.xhtml

20. Islam, M.M., et al., *Standardised alcohol screening in primary health care services targeting Aboriginal and Torres Strait Islander peoples in Australia.* Addiction Science & Clinical Practice, 2018. **13:5**.
21. Gray, D., et al., *Barriers and enablers to the provision of alcohol treatment among Aboriginal Australians: A thematic review of five research projects.* Drug and Alcohol Review, 2014.
22. Calabria, B., et al., *Identifying Aboriginal-specific AUDIT-C and AUDIT-3 cutoff scores for at-risk, high-risk, and likely dependent drinkers using measures of agreement with the 10-item Alcohol Use Disorders Identification Test.* Addiction Science & Clinical Practice, 2014. **9**(1): p. 17.
23. Australian Institute of Health and Welfare, *AIHW National Key Performance Indicators Database User guide reporting period ending 30 June 2017.* 2017.
24. Department of Health and Ageing, *Alcohol Treatment Guidelines for Indigenous Australians*. 2007, Commonwealth of Australia: Canberra.
25. National Health and Medical Research Council, *The NHMRC Road Map II: A strategic framework for improving the health of Aboriginal and Torres Strait Isalnder people through research.* 2010.
26. Australian Bureau of Statistics, *1216.0 - Australian Standard Geographical Classification (ASGC) - 2006* 2006.
27. Australian Bureau of Statistics, *Census of Population and Housing: Characteristics of Aboriginal and Torres Strait Islander Australians, 2011* 2076.0 2012, Canberra: ABS.
28. Panaretto, K., et al., *Evaluating performance of and organisational capacity to deliver brief interventions in Aboriginal and Torres Strait Islander medical services.* Australian and New Zealand journal of public health, 2010. **34**(1): p. 38-44.
29. Chikritzhs, T. and M. Brady, *Fact or fiction? ?A critique of the National Aboriginal and Torres Strait Islander Social Survey 2002.* Drug and Alcohol Review, 2006. **25**(3): p. 277-287.
30. Britt, H., et al., *General practice in Australia, health priorities and policy 1998 to 2008.* General practice series no. 24; Cat. no. GEP 24. 2009, Canberra: AIHW.
31. Sanatinia, R., et al., *Brief intervention for alcohol misuse in people attending sexual health clinics: study protocol for a randomized controlled trial.* Trials, 2012. **13**(1): p. 1-8.
32. Coulton, S., et al., *Screening and brief interventions for hazardous alcohol use in accident and emergency departments: a randomised controlled trial protocol.* BMC health services research, 2009. **9**(1): p. 114.
33. Carey M, B.H., Sanson-Fisher R, *The cycle of change: implementing best-evidence clinical practice.* International Journal for Quality in Health Care, 2009. **21**(1): p. 37-43.
34. Kowalyszyn, M. and A. Kelly, *Family functioning, alcohol expectancies and alcohol-related problems in a remote Aboriginal Australian community: a preliminary psychometric validation study.* Drug and Alcohol Review, 2003. **22**(1): p. 53-59.
35. Lee, K., et al., *Handbook for Aboriginal Alcohol and Drug Work*. 2012, Sydney, NSW: University of Sydney.
36. Haber, P., et al., *Quick reference guide to the treatment of alcohol problems: companion document to the guidelines for the treatment of alcohol problems*. 2009, Prepared for the Australian Government Commonwealth Department of Health and Ageing: Canberra. p. 9.
37. Hirsch, A., et al., *Every door closed: Barriers facing parents with criminal records*. 2002, Washington: Community Legal Services, Inc.
38. Ministerial Council on Drug Strategy, *National Drug Strategy Aboriginal and Torres Strait Islander Peoples Complementary Action Plan 2003-2009 (Background Paper)*. 2006, Commonwealth of Australia: Canberra.
39. Sibthorpe BM, et al., *The demise of a planned randomised controlled trial in an urban Aboriginal medical service.* Medical Journal of Australia, 2002. **176**(6): p. 248-9.

For peer review only - http://bmjopen.bmj.com/site/about/guidelines.xhtml

40. NSW Health Department, *A Framework for Building Capacity to Improve Health.* 2001.
41. Melanie Birks, Y.C., Karen Francis, *Memoing in qualitative research. Probing data and processes.* Journal of Research in Nursing, 2008. **13**(1): p. 68–75.
42. Morse, J.M. and P.A. Field, *Qualitative research methods for health professionals.* 1995. **2nd edition**: p. 254
43. Glaser, B. and A. Strauss, *The discovery of grounded theory: strategies for qualitative research.* Chicago: Aldine Publishing Company, 1967.

For peer review only

For peer review only - http://bmjopen.bmj.com/site/about/guidelines.xhtml

Figure Legends

Figure 1. Project principles – embedding mainstream evidence into a cultural care approach

Figure 2. Flow diagram for the trial

Figure 3. Three elements used to feedback alcohol screening data to ACCHSs

Figure 4: Project Timeline: SPIRIT schedule of enrolment, interventions, and assessments for the trial

For peer review only

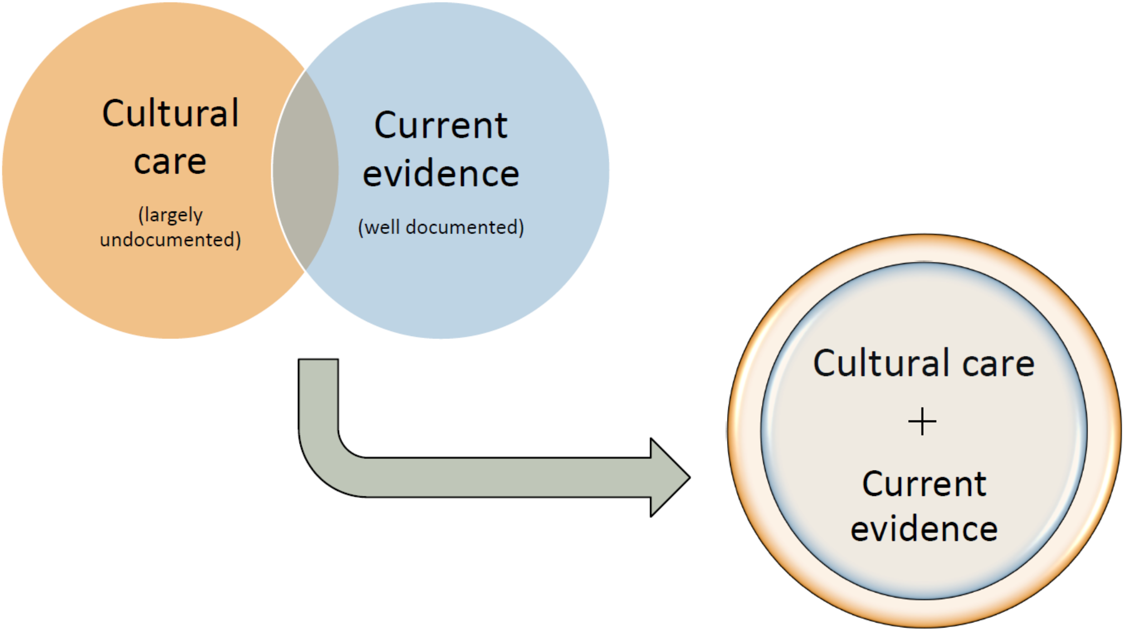
Cultural care
(largely undocumented)
Current evidence
(well documented)
Cultural care
+
Current evidence

review only

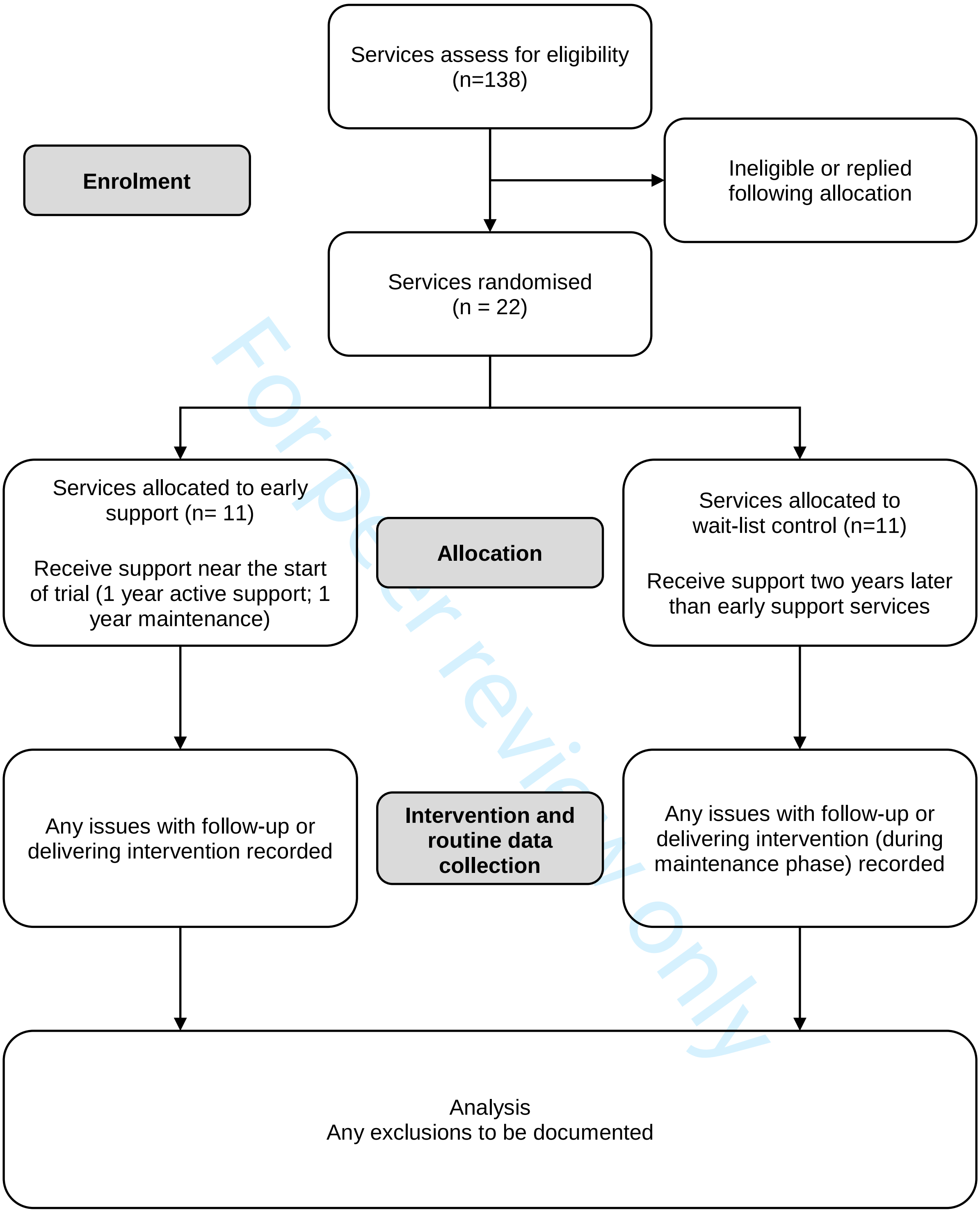

For peer review only

1. Are you meeting your AUDIT-C screening goal?

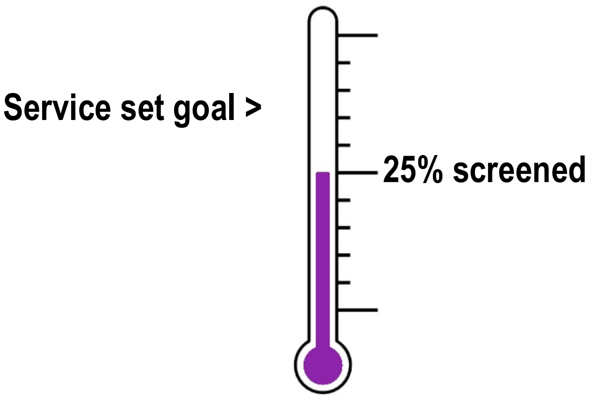

2. How many clients your service is screening

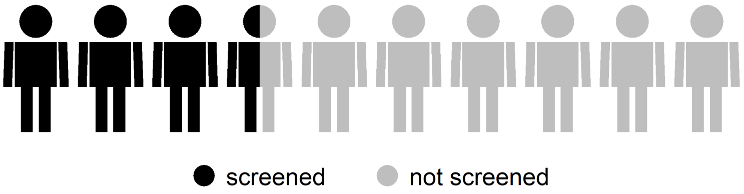

Screening rate over 12 months (out of clients aged 16+ who have attended in the past year)

3. How is your AUDIT-C screening rate going over time?

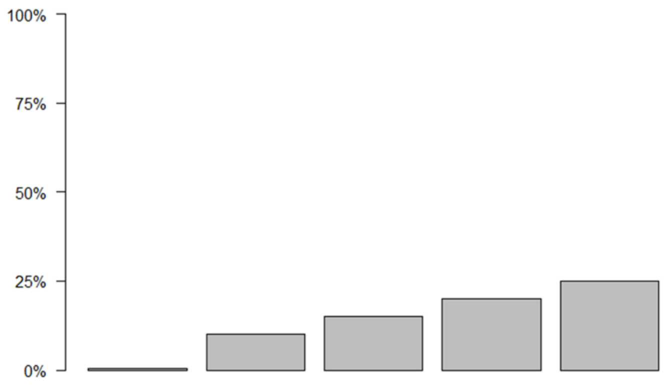

Screening rate for each 2-month period

Figure 4: Project Timeline: SPIRIT schedule of enrolment, interventions, and assessments for the trial

	Study period						
	**Enrolment**	**Allocation**	**Post-allocation**				
**TIMEPOINT**	***t_{-1} (Feb 16) Oct 2016 to Jun 2017***	**16/06/17**	***t_0 29/8/17 Year 1***	***t_1 28/8/18 Year 2***	***t_2 14/8/19 Year 3***	***t_3 13/8/20 Year 4***	***Close out 28/4/21***
**ENROLMENT:**							
Eligibility screen	X						
Informed consent	X						
Allocation		X					
**INTERVENTIONS:**							
Early support services			α	β.			
Late support services (wait-list control)					α	β.	
**ASSESSMENTS:**							
**Baseline/outcome variables** Retrospectively collected for 2-monthly periods† from Feb 2016 to Feb 2021: AUDIT-C screening rate; provision of brief interventions‡, counselling; prescriptions for acamprosate, naltrexone, disulfiram; AUDIT-C results; systolic BP; GGT; HbA1c§	X	X	X	X	X	X	X

*Data will be retrospectively extracted for the 12-month period before the start of the support (the intervention). All data used in this study are routinely collected clinical data, extracted from the practice software ('Communicare')

† Data will be extracted on the 28th day of every second month.

The first census date (t_1) is one year after the start of the intervention; $t_{2\text{-}4}$ are other census points.

‡ Unhealthy alcohol use = hazardous consumption or alcohol use disorders

For peer review only

§ Blood test results (GGT, HbA1c) are only available when these have been routinely conducted
α Active support phase
β Maintenance support phase

For peer review only

For peer review only

Beth Hummerston
Project Officer
Aboriginal Health Council of South Australia

Kristie Harrison Project Coordinator / PhD Candidate
Sydney Medical School, University of Sydney

Kate Conigrave FAChAM, FAFPHM, PhD
Senior Staff Specialist, Royal Prince Alfred Hospital
and Professor, Addiction Medicine, University of Sydney

[DATE]

name, CEO
organisation
address

Dear <Name>,

Re: Opportunity for your service to be supported further around alcohol management - a whole of service approach.

On behalf of our project team, I am excited to write to you to regarding an opportunity to take part in a national project. The project's goal is to make it easier for services to put in place evidence-based management of unhealthy alcohol use.

This project is the first of its kind in supporting Aboriginal & Torres Strait Islander (Indigenous) primary care services in making use of the full range of alcohol management approaches within their services.

We have recently been awarded an NHMRC grant for five years to undertake this project and to evaluate how well this support works. The project team will support services to refine their system-wide approaches to caring for individuals with unhealthy alcohol use based to their own service needs and on best evidence. There will also be sharing of learnings between services.

We know that alcohol can have very harmful effects on our communities and also that drinking even a little over recommended limits can increase the chance of cancers and other diseases. Taking a whole of service approach, which is critical to Indigenous values in addressing health issues, can have greater positive outcomes for patients and communities.

Please find attached a one page overview for more information about the project and the project team.

If you would like to discuss further please do not hesitate to give me or Kate Conigrave a call.

Warm regards,

Beth Hummerston	Kate Conigrave	Kristie Harrison
Project Officer	Senior Staff Specialist and Professor	Aboriginal Project Coordinator

[contact details provided for each signatory: land line, mobile and email address]

For peer review only - http://bmjopen.bmj.com/site/about/guidelines.xhtml

Information sheet for services

Prof Kate Conigrave
Drug Health Services, RPA Hospital
Missenden Rd
Camperdown NSW 2050
Email: kate.conigrave@sydney.edu.au Ph XXXXXXXX

Project: "Supporting Indigenous primary care services to reduce the harms from alcohol"
Information sheet: ***This is for you to keep***

Researchers: Kate Conigrave, Paul Haber, Noel Hayman, Kylie Lee, Rowena Ivers, Tim Dobbins, Dennis Gray, David Johnson, Scott Wilson

Institutions: Sydney and Curtin Universities; Inala Indigenous Health Service; Royal Prince Alfred Hospital; Illawarra Aboriginal Medical Service; University of NSW; Aboriginal Health Council SA and Aboriginal Drug and Alcohol Council SA.

Funding: NHMRC (Project Grant, started June 2016).

Why does this research need to be done?

- Drinking problems have a big impact on families and communities. Also, people who drink even a little above recommended limits can have a greater chance of cancer and other medical conditions.
- In primary care services, unhealthy drinking is not addressed as often as smoking. Health staff may feel less comfortable in talking about alcohol and less confident in using alcohol treatments (like medicines to help a person stay dry, or to treat withdrawal).
- Treatments for unhealthy drinking can be used in primary care, and have been shown to be effective in improving health. They are part of national alcohol treatment guidelines.

How is this research going to be done?

- We will test ways to support Aboriginal and Torres Strait Islander (Indigenous) primary care services to do the best they can to help people with unhealthy alcohol use. Approaches will be led by the services and guided by the evidence base.
- Twenty two primary care services around Australia will be invited to take part. To be involved, services will have: 1000+ clients in a 12-month period and use Communicare. Services can pull out at any time.
- Half the services (randomly selected) will receive support over two years to improve the way their service works with alcohol use – 'initial support' services. The other 11 services will wait to get this support at the end of the study – 'wait-control' services.
- The 11 'initial support' services will each choose two project 'champions' e.g. a clinician and a manager. The project coordinator will support those champions and their services to find and address barriers to addressing unhealthy drinking.
- To help this process, the project staff will interview up to five staff members from each service (including the champions) about barriers to change, and opportunities to address these barriers.

For peer review only - http://bmjopen.bmj.com/site/about/guidelines.xhtml

Information sheet for services

- The representatives of the 'initial support' services will join forces and share ideas and experiences in service improvement through a face-to-face workshop and second monthly teleconferences over a two-year period.
- Practical support will be provided to services during the support phase, including educational resources, on-site staff training, support with refining Communicare and support for developing or adapting resources. Agreed expenses will be reimbursed up to $9000 for 'initial support' services and $3500 for 'wait-control' services.
- Each 'initial support' service will get regular (four times a year) feedback on how they are going based on their Communicare data: how often they screen for unhealthy alcohol use and how often they provide advice or treatment. De-identified data will be transferred to an agreed secure and confidential location.
- Results will be compared between 'initial support' and 'wait-control' services on how often screening is done and how often treatment is provided. Any changes in service-wide levels of drinking, blood pressure and liver blood tests will also be noted.

Storage and use of the data
The data will be stored at the Aboriginal Health Council SA (AHCSA) and at the University of Sydney. The data will be used to give feedback to each service on how they are going. Also reports on the overall study will be prepared for services and for publication.

Confidentiality
Any information given to the researchers will only be used for this research, and no one outside the project team will have access to the raw data. The information will not be given out to other people, except in summary reports as we describe in this information sheet. No staff member will be able to be identified in any summary report. Services will not be named and efforts will be made to avoid revealing the identity of the service in any way. Services will be given the opportunity to comment on publications.

Voluntary nature of this study
Services can choose if they would like to take part in this study. Also, services can withdraw at any time, and without affecting their relationship with the researchers.

Risks and benefits of taking part
- The study is designed to take as little time of service staff as possible, and to maximize the benefit to the services.
- Services who take part will be assisted to help their clients work towards a healthy approach to alcohol. Staff should gain skills, knowledge and confidence.
- The study should provide a model for how to support other Indigenous primary care services to give the best possible care for unhealthy drinking within their communities

Contacts
For further information, please contact Prof Kate Conigrave (details above) or Kristie Harrison on *[phone number]* or on *[email address]*
If you have any concerns or complaints about this study you can contact Kate or Kristie, or the Menzies Human Ethics Research Committee (ph 08 8946 8600 or email ethics@menzies.edu.au):

For peer review only - http://bmjopen.bmj.com/site/about/guidelines.xhtml

Prof Kate Conigrave
Drug Health Services, RPA Hospital
Missenden Rd
Camperdown NSW 2050
Email: kate.conigrave@sydney.edu.au Ph 02 XXXX XXXX

Project: "Supporting Indigenous primary care services to reduce the harms from alcohol"

Consent Form: *This means you can say NO*

Project: Supporting Indigenous primary care services to reduce the harms from alcohol

Researchers: Kate Conigrave, Paul Haber, Noel Hayman, Kylie Lee, Rowena Ivers, Tim Dobbins, Dennis Gray, David Johnson, Scott Wilson

---

On behalf of **>Service Name<** , I freely give consent to participate in the above research project.

- ☐ I confirm that I have informed all relevant staff, and the Board of Directors about the project.
- ☐ I understand how the project will be conducted as described by members of the research team.
- ☐ I have received the project information sheet and have kept a copy of this at our service.
- ☐ I understand that our service can withdraw from the project at any time without reason and that this will not affect our relationship with the researchers.
- ☐ I consent to our service/staff:
  - Collaborating with the research team to develop a memorandum of understanding (MOU)
  - Committing to a whole of service approach for the project
  - Nominating two staff members (e.g. one managerial and one clinical) to champion the project
  - Participating in consultations, interviews, teleconferences and meetings to assist in the development and implementation of quality improvement approaches for our service.
- ☐ I authorise the extraction and transfer of de-identified patient data from **>Service Name<** Communicare system to researchers. I understand that these data will be kept secure in an agreed location.
- ☐ I understand that any information provided by staff of this service (e.g. in interviews or workshop) will be de-identified before being reported on publically and that the service will not be named in any publication.
- ☐ I confirm that I have had enough time to ask questions or request further information.

For peer review only - http://bmjopen.bmj.com/site/about/guidelines.xhtml

Consent form – Services-NT

CEO/Service representative Signature: ______________________ Date: _________ Time_____

Name: ____

Chairperson: Signature ____________________________________Date:________________

Name: ____

Service address: ____

Phone: ____

Fax: ____

Researcher Signature:_______________________________ Date: ______________________

Name: ____

Contacts: if you have any questions about this research please contact Kate Conigrave (details above) or Beth Hummerston via *[email address]* or *[phone number]*.

For peer review only - http://bmjopen.bmj.com/site/about/guidelines.xhtml

For peer review only

Information sheet for staff member interview

Prof Kate Conigrave
Drug Health Services, RPA Hospital
Missenden Rd
Camperdown NSW 2050
Email: kate.conigrave@sydney.edu.au Ph 02 XXXX XXXX

Project: "Supporting Indigenous primary care services to reduce the harms from alcohol"
This is for you to keep

Researchers: Kate Conigrave, Paul Haber, Noel Hayman, Kylie Lee, Rowena Ivers, Tim Dobbins, Dennis Gray, David Johnson, Scott Wilson

Institutions: Sydney and Curtin Universities; Inala Indigenous Health Service; Royal Prince Alfred Hospital; Illawarra Aboriginal Medical Service; University of NSW; Aboriginal Health Council SA and Aboriginal Drug and Alcohol Council SA.

Funding: NHMRC (Project Grant, started June 2016).

Why does this research need to be done?

- Drinking problems have a big impact on families and communities. Also, people who drink even a little above recommended limits can have a greater chance of cancer and other medical conditions.
- In primary care services, unhealthy drinking is not addressed as often as smoking. Health staff may feel less comfortable in talking about alcohol and less confident in using alcohol treatments (like medicines to help a person stay dry, or to treat withdrawal).
- Treatments for unhealthy drinking can be used in primary care, and have been shown to be effective in improving health. They are part of national alcohol treatment guidelines.

How is this research going to be done?

- Your service has agreed to take part in this study. The project will test ways of supporting Aboriginal and Torres Strait Islander (Indigenous) primary care services to prevent and treat alcohol problems. Approaches will be led by the services and guided by the evidence bbase.
- Twenty two primary care services around Australia are invited to take part.
- Half the services (randomly selected) will receive support over two years to improve the way their service works with alcohol use – 'initial support' services. The other 11 services will wait to get this support at the end of the study – 'wait-control' services. Your service was selected to receive the support early in the project.
- A range of practical support can be provided to all services. This can include educational resources, on-site staff training, support with refining Communicare and support for developing or adapting resources. .Services themselves will have ideas on what support would be useful.
- We invite 2-5 staff members from each 'initial support' service to take part in an interview. This interview is to share ideas on what are challenges in preventing and caring for alcohol problems, and what types of support might be useful in making it easier. The interview can also cover things that are working well in the way your service works in relation to alcohol.
- You are invited to take part in that staff member interview and share your ideas with us. The interview should last about 45 minutes.

For peer review only - http://bmjopen.bmj.com/site/about/guidelines.xhtml

Information sheet for staff member interview

- We will take notes on these interviews, and then make a summary of what we have learned from speaking with staff of the 11 'initial support services'.

Use of the data

We will use the summary report from the interviews at a workshop attended by representatives of the 11 'initial support' services. The summary will help guide the discussion and allow sharing of ideas. It will also help guide the support we provide over the rest of the project. We will also use the information from the interviews and workshop to write a summary report to help other services around Australia. We would share that summary through conferences or a scientific publication.

Confidentiality

The report will only have summary data and we will not identify an individual in any report or discussion.. Sometimes we will quote what a staff member said, but we will not name them or their service. Care will be taken that no staff member or service will be able to be identified in any report.. Services will be given the opportunity to provide comment on publications.

It is your choice if you take part

It is your choice if you take part in this interview. You can withdraw at any time, and that is fine. If you want us to, we can take your data out of the study.

Risks and benefits of taking part?

- We hope that you will enjoy taking part and that you will find it helpful in reflecting on how your service is working on the issue of alcohol
- There is minimal risk in taking part, as the results of the interview are confidential and you are not identified in the summary report.
- The interview will help to inform what support your service and other support services get from the project team. This support should make it easier for your service to help clients to have a healthy approach to alcohol.
- The study should provide a model for how to support Indigenous primary care services to give the best possible care for unhealthy drinking within their communities.

Contacts

For further information, please contact Prof Kate Conigrave (details above) or Beth Hummerston via *[email address]* or *[phone number]*

If you have any concerns or complaints about this study you can contact Kate or Beth, or the Menzies Human Ethics Research Committee (ph 08 8946 8600 or email ethics@menzies.edu.au):

For peer review only

For peer review only - http://bmjopen.bmj.com/site/about/guidelines.xhtml

Consent for interview – Staff member

THE UNIVERSITY OF SYDNEY

Prof Kate Conigrave FAChAM, FAFPHM, PhD
Royal Prince Alfred Hospital
Missenden Rd,
Camperdown NSW 2050
Ph: 02 9515 8650 FAX: 02 9515 5779 Email: kate.conigrave@sydney.edu.au

Project: Supporting Indigenous primary care services to reduce the harms from alcohol

Consent for interview
"This means you can say NO"

Researchers: Kate Conigrave, Paul Haber, Noel Hayman, Kylie Lee, Rowena Ivers, Tim Dobbins, Dennis Gray, David Johnson, Scott Wilson

I confirm that:

- ☐ I have received information about this research project.
- ☐ The research project has been explained to me and I fully understand why the research is being done and what I am being asked to do in it.
- ☐ I understand that I may stop taking part in this research interview at any stage.
- ☐ I understand that I may not directly benefit from taking part in the project.
- ☐ I understand that while information gained during the study may be published, I will not be identified in any way.

Signature: ________________________________Date: ________________Time_______

Name of staff member: ____

I have explained the research project to the participant and believe that he/she understands what is involved.

Researcher's signature: ____ Date: _________________

Name of researcher: ____

Contacts: if you have any questions about this research please contact Kate Conigrave (details above) or Beth Hummerston via beth.hummerston@ahcsa.org.au or (08) 8273-7200.

For peer review only - http://bmjopen.bmj.com/site/about/guidelines.xhtml

Aboriginal Health Council of South Australia

THE UNIVERSITY OF SYDNEY

Reducing Harms from Alcohol
Alcohol Treatment Project - Communicare Indicators

Data extraction and storage

Communicare data is extracted by a series of 11 SQL reports. These reports are uploaded to the practice software at each site.

[details of how to send the data to University of Sydney omitted here for brevity]

The service name is not recorded on the data base. Instead a number from 1 to 22 is used to identify a folder for the data for each service.

Each patient within a service is identified by a Communicare ID. These numbers are not unique BETWEEN services, only within services. No name, address , date of birth or other identifying data is collected.

Key variables being extracted

1. **Responses to each of the three questions within the clinical item *Check up;alcohol;AUDIT-C***
   - Q1: How often do you have a drink containing alcohol?
   - Q2: How many drinks containing alcohol do you have on a typical day when you are drinking?
   - Q3: How often do you have six or more drinks on one occasion?

   Information within each of these reports; Patient ID, Aboriginality, Age, Date the qualifier was recorded, the clinical item in which the qualifier was recorded, and the name of the qualifier and the qualifier response.

2. **Drinking status extracted as Communicare qualifier *Alcohol consumption level***
   - Status is recorded as 'Ex-drinker', 'Non-drinker', 'Unsafe – needs intervention', or, 'Within safe drinking limits'

   Information within each of these reports includes: Patient ID, Age, Gender, Date the qualifier was recorded, clinical item in which the qualifier was recorded in.

3. **Non pharmaceutical 'treatment' provided for unhealthy drinking:**

   Two relevant clinical items that capture alcohol 'treatment' (unless Communicare has been locally modified):
   - ***Advice/education; alcohol***; and
   - ***Counselling; alcohol***

   Information within each of these reports includes: Patient ID, Aboriginality, Age, Gender, Date the qualifier was recorded, and the clinical item in which the qualifier was recorded.

4. **Alcohol relapse prevention medicines:**

   A list of scripts for alcohol relapse prevention meds (searching for both brand and generic names of the medicines acamprosate, naltrexone and disulfiram.

   Information within each of these reports includes; Patient ID, Client status, Aboriginality, Age, Gender, Generic drug name, Prescription details, Prescription date

5. **Biomedical results: GGT, systolic BP and HbA1C**

   Information within each of these reports; Patient ID, Age, Gender, Date the result was recorded, Clinical item the result was record in, qualifier name the result was recorded in, result.

For peer review only - http://bmjopen.bmj.com/site/about/guidelines.xhtml

SPIRIT
STANDARD PROTOCOL ITEMS: RECOMMENDATIONS FOR INTERVENTIONAL TRIALS

SPIRIT 2013 Checklist: Recommended items to address in a clinical trial protocol and related documents*

Section/item	Item No	Description	Addressed on page number
**Administrative information**			
Title	1	Descriptive title identifying the study design, population, interventions, and, if applicable, trial acronym	i
Trial registration	2a	Trial identifier and registry name. If not yet registered, name of intended registry	iv; Table 2 (p13)
	2b	All items from the World Health Organization Trial Registration Data Set	Table 2 (p13)
Protocol version	3	Date and version identifier	iv
Funding	4	Sources and types of financial, material, and other support	15
Roles and responsibilities	5a	Names, affiliations, and roles of protocol contributors	i, 15
	5b	Name and contact information for the trial sponsor	15
	5c	Role of study sponsor and funders, if any, in study design; collection, management, analysis, and interpretation of data; writing of the report; and the decision to submit the report for publication, including whether they will have ultimate authority over any of these activities	15
	5d	Composition, roles, and responsibilities of the coordinating centre, steering committee, endpoint adjudication committee, data management team, and other individuals or groups overseeing the trial, if applicable (see Item 21a for data monitoring committee)	i, 15

For peer review only

**Introduction**			
Background and rationale	6a	Description of research question and justification for undertaking the trial, including summary of relevant studies (published and unpublished) examining benefits and harms for each intervention	1 - 3
	6b	Explanation for choice of comparators	2
Objectives	7	Specific objectives or hypotheses	3
Trial design	8	Description of trial design including type of trial (eg, parallel group, crossover, factorial, single group), allocation ratio, and framework (eg, superiority, equivalence, noninferiority, exploratory)	3
**Methods: Participants, interventions, and outcomes**			
Study setting	9	Description of study settings (eg, community clinic, academic hospital) and list of countries where data will be collected. Reference to where list of study sites can be obtained	3
Eligibility criteria	10	Inclusion and exclusion criteria for participants. If applicable, eligibility criteria for study centres and individuals who will perform the interventions (eg, surgeons, psychotherapists)	4
Interventions	11a	Interventions for each group with sufficient detail to allow replication, including how and when they will be administered	6 – 9; Figure 2 (p3)
	11b	Criteria for discontinuing or modifying allocated interventions for a given trial participant (eg, drug dose change in response to harms, participant request, or improving/worsening disease)	12
	11c	Strategies to improve adherence to intervention protocols, and any procedures for monitoring adherence (eg, drug tablet return, laboratory tests)	10
	11d	Relevant concomitant care and interventions that are permitted or prohibited during the trial	N/A
Outcomes	12	Primary, secondary, and other outcomes, including the specific measurement variable (eg, systolic blood pressure), analysis metric (eg, change from baseline, final value, time to event), method of aggregation (eg, median, proportion), and time point for each outcome. Explanation of the clinical relevance of chosen efficacy and harm outcomes is strongly recommended	4
Participant timeline	13	Time schedule of enrolment, interventions (including any run-ins and washouts), assessments, and visits for participants. A schematic diagram is highly recommended (see Figure)	6, Figures 2 (p3)

For peer review only - http://bmjopen.bmj.com/site/about/guidelines.xhtml

Sample size	14	Estimated number of participants needed to achieve study objectives and how it was determined, including clinical and statistical assumptions supporting any sample size calculations	5
Recruitment	15	Strategies for achieving adequate participant enrolment to reach target sample size	4
**Methods: Assignment of interventions (for controlled trials)**			
Allocation:			
Sequence generation	16a	Method of generating the allocation sequence (eg, computer-generated random numbers), and list of any factors for stratification. To reduce predictability of a random sequence, details of any planned restriction (eg, blocking) should be provided in a separate document that is unavailable to those who enrol participants or assign interventions	5
Allocation concealment mechanism	16b	Mechanism of implementing the allocation sequence (eg, central telephone; sequentially numbered, opaque, sealed envelopes), describing any steps to conceal the sequence until interventions are assigned	5
Implementation	16c	Who will generate the allocation sequence, who will enrol participants, and who will assign participants to interventions	4-5
Blinding (masking)	17a	Who will be blinded after assignment to interventions (eg, trial participants, care providers, outcome assessors, data analysts), and how	6
	17b	If blinded, circumstances under which unblinding is permissible, and procedure for revealing a participant's allocated intervention during the trial	N/A
**Methods: Data collection, management, and analysis**			
Data collection methods	18a	Plans for assessment and collection of outcome, baseline, and other trial data, including any related processes to promote data quality (eg, duplicate measurements, training of assessors) and a description of study instruments (eg, questionnaires, laboratory tests) along with their reliability and validity, if known. Reference to where data collection forms can be found, if not in the protocol	9-10
	18b	Plans to promote participant retention and complete follow-up, including list of any outcome data to be collected for participants who discontinue or deviate from intervention protocols	10

For peer review only - http://bmjopen.bmj.com/site/about/guidelines.xhtml

Data management	19	Plans for data entry, coding, security, and storage, including any related processes to promote data quality (eg, double data entry; range checks for data values). Reference to where details of data management procedures can be found, if not in the protocol	10
Statistical methods	20a	Statistical methods for analysing primary and secondary outcomes. Reference to where other details of the statistical analysis plan can be found, if not in the protocol	11-12
	20b	Methods for any additional analyses (eg, subgroup and adjusted analyses)	11-12
	20c	Definition of analysis population relating to protocol non-adherence (eg, as randomised analysis), and any statistical methods to handle missing data (eg, multiple imputation).	12
**Methods: Monitoring**			
Data monitoring	21a	Composition of data monitoring committee (DMC); summary of its role and reporting structure; statement of whether it is independent from the sponsor and competing interests; and reference to where further details about its charter can be found, if not in the protocol. Alternatively, an explanation of why a DMC is not needed **It's mainly used where treatment can cause harms, here minimal risk**	12
	21b	Description of any interim analyses and stopping guidelines, including who will have access to these interim results and make the final decision to terminate the trial	N/A
Harms	22	Plans for collecting, assessing, reporting, and managing solicited and spontaneously reported adverse events and other unintended effects of trial interventions or trial conduct	10
Auditing	23	Frequency and procedures for auditing trial conduct, if any, and whether the process will be independent from investigators and the sponsor	12
**Ethics and dissemination**			
Research ethics approval	24	Plans for seeking research ethics committee/institutional review board (REC/IRB) approval	12
Protocol amendments	25	Plans for communicating important protocol modifications (eg, changes to eligibility criteria, outcomes, analyses) to relevant parties (eg, investigators, REC/IRBs, trial participants, trial registries, journals, regulators)	14

Consent or assent	26a	Who will obtain informed consent or assent from potential trial participants or authorised surrogates, and how (see Item 32)	4-5
	26b	Additional consent provisions for collection and use of participant data and biological specimens in ancillary studies, if applicable	5
Confidentiality	27	How personal information about potential and enrolled participants will be collected, shared, and maintained in order to protect confidentiality before, during, and after the trial. **do we need to mention data destroyed 7 years after last publication)?	10
Declaration of interests	28	Financial and other competing interests for principal investigators for the overall trial and each study site	15
Access to data	29	Statement of who will have access to the final trial dataset, and disclosure of contractual agreements that limit such access for investigators	10
Ancillary and post-trial care	30	Provisions, if any, for ancillary and post-trial care, and for compensation to those who suffer harm from trial participation	N/A
Dissemination policy	31a	Plans for investigators and sponsor to communicate trial results to participants, healthcare professionals, the public, and other relevant groups (eg, via publication, reporting in results databases, or other data sharing arrangements), including any publication restrictions	13
	31b	Authorship eligibility guidelines and any intended use of professional writers	13
	31c	Plans, if any, for granting public access to the full protocol, participant-level dataset, and statistical code	13
**Appendices**			
Informed consent materials	32	Model consent form and other related documentation given to participants and authorised surrogates	Appendices 1-4
Biological specimens	33	Plans for collection, laboratory evaluation, and storage of biological specimens for genetic or molecular analysis in the current trial and for future use in ancillary studies, if applicable	NA

*It is strongly recommended that this checklist be read in conjunction with the SPIRIT 2013 Explanation & Elaboration for important clarification on the items. Amendments to the protocol should be tracked and dated. The SPIRIT checklist is copyrighted by the SPIRIT Group under the Creative Commons "Attribution-NonCommercial-NoDerivs 3.0 Unported" license.

For peer review only - http://bmjopen.bmj.com/site/about/guidelines.xhtml

BMJ Open

Supporting Aboriginal Community Controlled Health Services to deliver alcohol care: protocol for a cluster randomised controlled trial

Journal:	*BMJ Open*
Manuscript ID	bmjopen-2019-030909.R1
Article Type:	Protocol
Date Submitted by the Author:	07-Aug-2019

Complete List of Authors:	Harrison, Kristie; The University of Sydney, Faculty of Medicine and Health<br>Lee, KS; The University of Sydney, Faculty of Medicine and Health; La Trobe University, Centre for Alcohol Policy Research<br>Dobbins, Timothy; University of New South Wales, School of Public Health and Community Medicine<br>Wilson, Scott; Aboriginal Drug and Alcohol Council of South Australia, ; The University of Sydney, Faculty of Medicine and Health<br>Hayman, Noel; Southern Queensland Centre of Excellence in Aboriginal and Torres Strait Islander Primary Health Care (Inala Indigenous Health Service); Griffith University, School of Medicine<br>Ivers, Rowena; Illawarra Aboriginal Medical Service; The University of Sydney, Faculty of Medicine and Health<br>Haber, Paul; University of Sydney - Camperdown and Darlington Campus, Discipline of Addiction medicine; Sydney Local Health District, Drug Health Services<br>Conigrave, James; The University of Sydney, Faculty of Medicine and Health<br>Johnson, David; Aboriginal Health Council of South Australia<br>Hummerston, Beth; Aboriginal Health Council of South Australia,<br>Gray, Dennis ; Curtin University Bentley Campus, National Drug Research Institute<br>Conigrave, Katherine; University of Sydney, Sydney Medical School; Royal Prince Alfred Hospital, Drug Health Services

**Primary Subject Heading**:	Health services research
Secondary Subject Heading:	General practice / Family practice, Health services research, Public health, Evidence based practice, Addiction
Keywords:	Aboriginal, alcohol, screening, AUDIT-C, health services, PREVENTIVE MEDICINE

SCHOLARONE™
Manuscripts

Supporting Aboriginal Community Controlled Health Services to deliver alcohol care: protocol for a cluster randomised controlled trial

Kristie H Harrison[1]

KS Kylie Lee[2, 3]

Timothy Dobbins[4]

Scott Wilson[5, 2]

Noel Hayman[6, 7, 8]

Rowena Ivers[9, 1]

Paul S Haber[10, 2]

James H Conigrave[2]

David Johnson[11]

Beth Hummerston[11]

Dennis Gray[12]

Katherine M Conigrave[10, 2]

[1] The University of Sydney, Faculty of Medicine and Health, School of Public Health, NHMRC Centre of Research Excellence in Indigenous Health and Alcohol; C39, University of Sydney, New South Wales 2006 Australia

[2] The University of Sydney, Faculty of Medicine and Health, Discipline of Addiction Medicine, NHMRC Centre of Research Excellence in Indigenous Health and Alcohol; C39, University of Sydney, New South Wales 2006 Australia

[3] La Trobe University, Centre for Alcohol Policy Research, Bundoora, Victoria 3083 Australia

For peer review only

For peer review only - http://bmjopen.bmj.com/site/about/guidelines.xhtml

[4] University of New South Wales, National Drug and Alcohol Research Centre, Kensington, New South Wales 2052 Australia

[5] Aboriginal Drug and Alcohol Council Inc.,155 Holbrooks Rd, Underdale, South Australia 5032 Australia

[6] Southern Queensland Centre of Excellence in Aboriginal and Torres Strait Islander Primary Health Care, PO Box 52, Inala, Queensland 4077 Australia

[7] Griffith University, School of Medicine, Parklands Drive, Southport, Queensland 4215 Australia

[8] University of Queensland, School of Medicine, St Lucia, Queensland 4072 Australia

[9] Illawarra Aboriginal Medical Service, 150 Church St, Wollongong, New South Wales 2500 Australia

[10] Sydney Local Health District, Royal Prince Alfred Hospital, Drug Health Services, Missenden Rd, Camperdown, New South Wales 2050 Australia

[11] Aboriginal Health Council of South Australia, 220 Franklin St, Adelaide, South Australia 5000 Australia

[12] National Drug Research Institute, Curtin University, 7 Parker Place, Technology Park, Bentley Western Australia 6102 Australia

Corresponding author

Kate Conigrave

Drug Health Services, Royal Prince Alfred Hospital

Missenden Road

Camperdown NSW 2050

Phone +61 2 9515 8650 Email: kate.conigrave@sydney.edu.au

For peer review only

ABSTRACT

Introduction

Indigenous peoples who have experienced colonisation or oppression can have a higher prevalence of alcohol-related harms. In Australia, Aboriginal Community Controlled Health Services (ACCHSs) offer culturally accessible care to Aboriginal and Torres Strait Islander (Indigenous) peoples. However there are many competing health, socio-economic and cultural client needs.

Methods and analysis

A randomised cluster wait-control trial will test the effectiveness of a model of tailored and collaborative support for ACCHSs in increasing use of alcohol screening (with AUDIT-C) and of treatment provision (brief intervention, counselling or relapse prevention medicines).

Setting: Twenty-two ACCHSs across Australia

Randomisation: Services were stratified by remoteness, then randomised into two groups. Half receive support soon after the trial starts (intervention or 'early support'); half receive support two years later (wait-control or 'late support').

The Support: Core support elements will be tailored to local needs and include: support to nominate two staff as champions for increasing alcohol care; a national training workshop and bi-monthly teleconferences for service champions to share knowledge; onsite training, and bi-monthly feedback on routinely collected data on screening and treatment provision.

Outcomes and analysis: Primary outcome is use of screening using AUDIT-C as routinely recorded on practice software. Secondary outcomes are recording of brief intervention, counselling, relapse prevention medicines; and blood pressure, gamma glutamyltransferase and HbA1c. Multi-level logistic regression will be used to test the effectiveness of support.

For peer review only - http://bmjopen.bmj.com/site/about/guidelines.xhtml

For peer review only

Ethics and dissemination

Approval was obtained from eight ethics committees (Aboriginal-specific where available): in New South Wales (#1217/16); Central Australia (#CA-17-2842); Northern Territory (# 2017-2737); Central Queensland (#17/QCQ/9); Far North Queensland (#17/QCH/45-1143); South Australia (#04-16-694); St Vincents Hospital (Melbourne; #LRR 036/17); and Western Australian (#779). Results will be disseminated through reports to ACCHSs, peer-reviewed publications and conferences.

Trial registration

Australian New Zealand Clinical Trials Registry (ACTRN12618001892202 Version 1) retrospectively registered 21/11/18.

Strengths and limitations of this study

- This large cluster randomised controlled trial provides the power to test whether a model of support for Aboriginal and Torres Strait Islander primary care services can increase rates of alcohol screening and treatment provision.
- The protocol has been designed to be compatible with cultural context and to integrate western knowledge with the expertise and holistic approaches of these services.
- The use of regular data feedback and nomination of service champions offers the services an opportunity to be involved in ongoing quality improvement on alcohol care.
- The resultant study will be able to use routinely collected outcome data but this relies on the accurate recording of screening and alcohol care provided to clients.

For peer review only - http://bmjopen.bmj.com/site/about/guidelines.xhtml

INTRODUCTION

Globally, alcohol is the leading cause of death and disease for all people between the ages of 15-49 [1]. Indigenous peoples dealing with colonisation and oppression can be at higher risk of mental illness and harms from alcohol [2]. In Australia, the proportion of Aboriginal and Torres Strait Islander (Indigenous) people who drink alcohol is less than non-Indigenous people [3]. However, Indigenous Australians are 2-8 times more likely to be hospitalised for alcohol-related conditions [4] and nine times more likely to die from alcohol-related harms [5]. This directly impacts on the health gap between Indigenous and non-Indigenous Australians [4]. These increased risks and harms from drinking can be related to experiences of trauma, grief, poverty and cultural dispossession, resulting in increased mental illness, trans-generational trauma and lower life expectancy [2, 4, 6]. The lack of community autonomy and access to culturally appropriate treatment services also contribute [4].

Early detection of unhealthy drinking (i.e. of hazardous, harmful or dependent alcohol use) [7, 8] plays a key role in reducing the overall burden of alcohol conditions, particularly before people develop health problems or social impacts such as incarceration related to alcohol use [9-11]. Internationally, alcohol screening and brief intervention approaches have been found to be cost-effective in reducing unhealthy drinking, particularly for those who are not dependent on alcohol [9, 12]. However across Australian primary care settings, screening and brief intervention for unhealthy drinking is not always standardised [13], informed by Australian National Health and Medical Research Council (NHMRC) drinking guidelines [14], or systematically conducted. It is estimated that 50-70% of people with unhealthy drinking go undetected in Australian primary care [15].This percentage was the same (50-70% undetected) in four Indigenous primary care settings in Queensland, Australia (in 2001) [16]. Even when alcohol dependence is present, pharmacological treatments to reduce risk of relapse are not regularly prescribed in primary health care [17-19].

Culturally-specific health care services that are led and delivered by Indigenous peoples can have a key role in improving quality of healthcare and treatment access [20]. Aboriginal Community

For peer review only - http://bmjopen.bmj.com/site/about/guidelines.xhtml

Controlled Health Services (ACCHSs) provide accessible and culturally appropriate health care to Indigenous communities across Australia. Each ACCHS has its own holistic-based service delivery model for the communities that they serve [20]. As with mainstream (general population) services, knowledge and practice of screening and brief intervention varies between and within Indigenous primary care services [13, 21, 22]. Some ACCHSs have an alcohol and other drug (AOD) worker or team, or links with external AOD specialists/services. Others may have no specific AOD expertise and health practitioners may lack confidence in delivering alcohol care (i.e. screening, brief intervention, counselling or medicines). Prior to 2017, the Australian government had asked ACCHS staff to record clients' alcohol use as 'safe' or 'unsafe', 'non-drinker' or 'ex-drinker' [22]. However health practitioners may or may not have known current drinking guidelines or have applied these to assess the risk.

To increase standardisation of alcohol screening in ACCHSs, in July 2017 the use of AUDIT-C (the Alcohol Use Disorders Identification Test-Consumption) was added as a national key performance indicator for ACCHSs [23]. AUDIT-C [24] is a 3-item screening tool, comprised of the first three questions of the 10-item AUDIT [25]. This short form has been shown to have good sensitivity and specificity in comparison to the full AUDIT [24]. Several studies suggest that AUDIT-C is valid in Indigenous primary care settings, though further study into culturally appropriate delivery is suggested [26-28].

Internationally, research suggests that support can help primary care services increase implementation of alcohol screening and brief intervention [29] or provision of pharmacotherapies for relapse prevention in alcohol dependence [19]. Support which is multi-faceted, and targets several areas of a health service seems particularly promising [29]. For example the approach may target not just doctors, but other professionals, clients and organisational systems [29]. Continuing quality improvement approaches may offer sustainable benefit, including in ACCHSs [30]. However there have been very few studies of culturally-relevant support models for ACCHSs in alcohol care [21, 30], with even fewer conducted by Indigenous researchers.

For peer review only - http://bmjopen.bmj.com/site/about/guidelines.xhtml

For peer review only

This paper outlines the trial protocol for testing if a model of support can assist ACCHSs to integrate the mainstream evidence base on alcohol care with their own cultural and clinical expertise. The use of an inactive control group can be challenging on ethical and partnership grounds, particularly for vulnerable communities. In contrast, a wait-control design allows support to be delivered to all participating ACCHSs.

Aims

This trial aims to assess if a model of tailored and collaborative support for ACCHSs can result in increased uptake of evidence-based screening and treatment for unhealthy alcohol use (i.e. drinking above recommended limits). This service-wide model of support will be designed to build upon services' existing strengths (Figure 1).

(Insert Figure 1 about here)

METHODS

Overview

A cluster randomised trial will test the effectiveness of a model of tailored and collaborative support to increase the use of AUDIT-C and evidence-based treatment in ACCHSs compared with care as usual. Culturally relevant supports will be offered with the aim of increasing staff skills and capacity to develop, refine and deliver systems for alcohol care. These supports will be based on current evidence and be in-line with services' cultural-care practices. Twenty-two ACCHSs will be recruited across Australia. Services will be randomised into two arms, an early support (intervention) and a late support (wait-control) arm (Figure 2).

The support provided will contain eight core elements (Table 1), but be tailored, guided by ACCHSs, to their individual needs, including needs by remoteness. It will also be underpinned by current evidence, such as national alcohol treatment guidelines [10], Alcohol Treatment Guidelines for Indigenous Australians [31] and national alcohol consumption guidelines [14]. The study

For peer review only - http://bmjopen.bmj.com/site/about/guidelines.xhtml

methods will be based on principles of conducting health research with Indigenous Australians [32]. This includes Indigenous involvement and engagement, benefits for Indigenous peoples, and led by Indigenous Australians.

The primary outcome will be routinely collected data on screening rates using AUDIT-C. Secondary outcomes will include recorded delivery of brief intervention, counselling or prescribed medicines to reduce relapse in alcohol dependence. We will also describe changes in reported drinking and in biological measures that can be affected by drinking such as gamma glutamyltransferase (GGT, a liver enzyme), [33], HbA1C [34] and systolic blood pressure (BP) [34].

Participating services and eligibility

Recruitment

This trial will recruit whole services (no clients will be recruited). ACCHSs across Australia will be recruited based on expression of interest after circulation of study information to eligible services. A mix of services will be approached (by KH, BH or KC) with a range of remoteness as determined by the Australian Standard Geographical Classification – Remoteness Area (ASGC-RA) [35]. Both verbal and written information will be shared. Face-to-face meetings will be offered to eligible services. Indigenous project staff will be employed in recognition of their specialist skills and knowledge of working with ACCHSs. Additional time will be factored into project timelines for engaging with ACCHSs, including liaising with multiple key staff, follow-up conversations, and knowledge translation of research documents.

Inclusion criteria

Services eligible for the trial will meet the following criteria:

1. Are an ACCHS
2. Provide care to 1000+ clients per annum
3. Use Communicare™ as their practice software

For peer review only - http://bmjopen.bmj.com/site/about/guidelines.xhtml

Exclusion criteria

Services will only be excluded if signed institutional consent forms are not returned before the close of recruitment. Data from clients aged 15 or under will not be extracted.

Consent

After written institutional consent is signed, a memorandum of understanding (MOU) will be negotiated by the senior investigator (KC) and lead author (KH) with each ACCHS. Staff participating in individual qualitative interviews will also provide informed consent. Further consent will be sought, following completion of the main trial, if the authors wish to perform ancillary studies using stored data.

Randomisation

ACCHSs will be categorised into three strata based on their remoteness using ASGC-RA [35]: 1) urban and inner regional; 2) outer regional and remote; 3) very remote. Service names will be replaced by consecutive numbers. Within each stratum, half the services will be randomised by a computer program into the early support arm and half into the late support arm. Randomisation will be conducted by an author (TD) who is blind to services' names.

Sample size

Sample size requirements were calculated using PASS [36]. Because larger numbers are needed to be able to determine increases in treatment provision than to demonstrate an increase in screening, the treatment provision outcome was used for sample size calculation. In an ACCHS of 1000+ clients per year, approximately 60% (n=600) are likely to be aged 16 years or older [37]. We estimate that 57% of clients (n=342) have likely been screened for alcohol use within 12 months [38 and at least 25% (n=86) of screened clients are likely to be drinking above NHMRC recommended limits [14, 39, 40]. In the late support arm, it is likely that 60% (n=51) of identified unhealthy drinkers will have an alcohol intervention recorded [38]. Assuming an Intra-cluster correlation coefficient (ICC) of 0.04 [41, 42], enrolling 10 early support services and 10 late support services will allow for an increase in treatment provision over a 12-month period of at least 13% in the early support services

For peer review only - http://bmjopen.bmj.com/site/about/guidelines.xhtml

to be detected (i.e. from 60% to 73%; 80% power and 2-sided significance of 0.05). We will enrol an additional service in each arm to allow for the possibility of clusters dropping out of the study. Accordingly, the target number of services to be enrolled is 22.

The model of support

ACCHSs will be acknowledged as the experts in cultural health care for Indigenous peoples in their communities and Australia. The study also recognises the need for flexibility based on needs of individual services and of the ACCHS sector as a specialist sector overall. Support approaches will include the same core elements (Table 1). However, approaches will be tailored to the needs of each ACCHS, for what works best for their service, clients and communities.

Table 1. Eight core support elements*

**1.**	**Service champions**	Services asked to nominate two representatives to act as advocates of alcohol care and links between the service and research team
**2.**	**National workshop**	National capacity building workshop for service champions at the start of the support phase (and for early support services, a wrap-up feedback workshop at the end of the maintenance support phase)
**3.**	**Onsite training**	Training will be offered to each ACCHS at their service
**4.**	**Resources/funding**	Some resources (e.g. visual resources for brief intervention and clinical guidelines), will be given to services for free. Additional funding will be provided for the selection and purchase of further resources (e.g. FASD doll, standard drink cups).

For peer review only - http://bmjopen.bmj.com/site/about/guidelines.xhtml

**5.**	**Practice software support**	Support will be provided to services throughout the trial to facilitate the routine clinical use of alcohol-related items in the practice software.
**6.**	**Data feedback**	Individual service data will be fed back to each service every second month
**7.**	**Phone conferences**	Phone conferences will be held every second month between service champions and project team (including an addiction medicine physician). These will allow sharing of ideas and joint problem solving.
**8.**	**Online platform**	Further resources and information will be shared via a secure online platform.

*These eight core elements of support will be tailored to local service needs. Further detail on each element is provided in the body of the text.

The core support elements were designed based on evidence-based approaches for supporting implementation of alcohol care [21, 29]. The project team also drew on their experience working within (six authors) and with Aboriginal health services; and in health workforce development (nine authors). Advice was also received from ACCHS peak organisations and networks in NSW and SA, and from a research team conducting a quality improvement trial for ACCHSs on diabetes screening [43].

At the start of the early support phase, the support will be refined based on preliminary analysis of qualitative data from staff interviews and after feedback from the initial national workshop for early support service representatives. Tailoring and further minor refinement of support will occur during the trial, informed by service feedback.

Early support ('intervention') services will receive the support soon after the trial starts (one year of active support, one year maintenance phase). Late support (wait-control) services will receive the

For peer review only - http://bmjopen.bmj.com/site/about/guidelines.xhtml

same intervention elements two years after the start of the early-support phase. Due to the nature of the intervention, it is not possible to blind participants or staff as to which services are receiving support.

1. Identifying service champions

Each service will be asked to identify two staff representatives to work collaboratively with their service staff and the research team on increasing alcohol care. Services will be asked to consider nominating a clinician, Aboriginal health professional and/or an individual from management. Service champions will be invited to attend an initial national face-to-face workshop, and second-monthly teleconferences. These meetings will ensure that support approaches are in line with the values of each ACCHS, and will allow sharing of expertise and initiatives. Champions will be key in highlighting the potential benefits of change in their service and will encourage service staff to discuss their second monthly data feedback, and to address any key barriers to change [44].

2. Workshops

A national workshop will be held at the beginning of each support phase to bring champions from those services together for capacity building and networking. Feedback will be obtained from champions about pre- and post-workshop knowledge, training preferences, priority areas for support and useful resources. Key presentations from workshops will be video-recorded and made available via a password-protected website.

A wrap-up workshop will be organised for early support service champions (only) at the end of their support phase. Opportunities will be provided for service champions to provide verbal and written feedback on support, to discuss continuing quality improvement approaches and to network. Study results to that point will be discussed.

For peer review only - http://bmjopen.bmj.com/site/about/guidelines.xhtml

3. Onsite training

Training will be offered at each service for all staff, with maximum duration of two days. Core elements will include screening, alcohol and the body, brief intervention and treatment approaches, including relapse prevention medicines. Due to the individuality of each service, flexibility will be given to allow inclusion of other (alcohol-related) training topics. The training will be co-facilitated by an Addiction Specialist (KC) and an Aboriginal researcher with clinical background (KH) and will use culturally specific content and resources. Training content will be aligned with Indigenous protocols such as gender appropriateness, kinship systems and cultural obligations; such a family and community sharing, which at times can impact on substance use [27, 45]. Pre- and post-training feedback will be collected from participants.

4. Resources / Funding

The trial will provide several hard copy and electronic resources for free to services (i.e. the Handbook for Aboriginal Alcohol and Drug Work [46], Alcohol Awareness Kit [46] and Quick Reference Guide to the Treatment of Alcohol Problems [47]).

Each ACCHS will be provided with funding to purchase additional resources. Services can choose, in partnership with the chief investigators, how they spend their funding. The early support arm of the project will receive up to $9,000 for resources. The late support arm will receive up to $3,500. This is a reduced sum as they will have access to resources developed within the early support phase, so time and development costs will be less. Resources must be alcohol-related and may include additional training, conference attendance, education resources for staff or clients, funds for health promotion events or for local adaptation of resources.

5. Practice software support

A Communicare™ support officer will be available during the trial to support ACCHSs with the practice software. Some ACCHSs may choose to make modifications to Communicare™ to make alcohol 'clinical items' more accessible for staff. One example is adding AUDIT-C into the Aboriginal and Torres Strait Islander (Indigenous) health assessment ('Adult Health Check'),

For peer review only - http://bmjopen.bmj.com/site/about/guidelines.xhtml

which was not routine in standard versions of Communicare^{TM} at the time of trial commencement. AUDIT-C can also be added to other regular assessments such as: antenatal and the pre-consult examination (i.e. routine observations and/screening by a nurse or Aboriginal health worker before seeing the doctor). Services will also be provided with information about existing fields ('clinical items') available on Communicare^{TM} for recording episodes of alcohol care (education/advice; on-site counselling), which can then be retrieved by data extraction.

6. Second monthly data feedback

Individual services will receive feedback on their routinely collected data on alcohol care every two months during their support phase. Data will be represented visually using images and or graphs (Figure 3) and be fed back to nominated staff, including champions. Services will be asked for feedback on the data representation early in the support phase and adaptions will be made as requested. Services will be encouraged to discuss the results among staff, to see if there are ways to further improve alcohol screening and treatment rates.

(Insert Figure 3 about here)

7. Phone conferences

Second-monthly phone conferences will be organised for service champions. These will provide the opportunity for ACCHSs to network nationally. The champions will be split into two groups for the phone conferences, based on remoteness. During teleconferences, champions can discuss their data feedback, progress, successes, difficulties, helpful tools, resources or approaches.

8. Online platform

A password-protected web portal will allow ACCHS staff to access and share current evidence, culturally appropriate resources, and to network. It will include a discussion board. Short videos, training presentation slides, and clinical protocols will be uploaded by project staff.

For peer review only - http://bmjopen.bmj.com/site/about/guidelines.xhtml

Data collection

Qualitative data

Qualitative data will be collected from ACCHSs at the start of the early support arm. The data will help inform the support model. Structured phone interviews will be conducted with approximately five staff members from each ACCHS in the early support arm, with the aim of a total of fifty. Champions and/or key staff will be approached to assist with recruiting interviewees.

Alcohol can be sensitive to discuss due to cultural protocols such as kinship/family systems, gender or age, shame (embarrassment) or community and social acceptance of alcohol [48-50]. Therefore, the interview questions will be framed using a strengths-based, capacity-building approach [51]. Interview questions will include: approaches to make it easier to talk about alcohol, treatment around alcohol, skills and knowledge needed to provide quality alcohol care, new ways of helping people who struggle with alcohol, other successful programs that could be used as a model for alcohol care, and ideas for improvement in alcohol care.

Interviews will be conducted by two Indigenous staff with experience working with ACCHSs and conducting interviews. One interviewer will facilitate the interview while the other will live-transcribe. The interviewers will compare and discuss notes after each interview. Memos will also be written by the lead author (KH) on each interview.

Quantitative data

Non-identifiable data on episodes of screening and alcohol care and related variables (see below) will be extracted from the Communicare™ database of each ACCHS on the 28th day of every second month throughout the 5-year trial (Figure 4). The baseline extraction will include data for 12 months before the trial starts. Participating services use Structured Query Language (SQL) queries developed for this project to extract routinely collected data. For every 2-month period a denominator sheet will be extracted which includes the date of the last visit for each client who has attended during that period and their gender and age. In addition the following variables will be extracted, each one linked to an individual client ID and the date the variable was recorded:

For peer review only - http://bmjopen.bmj.com/site/about/guidelines.xhtml

AUDIT-C responses; the clinicians' perception of the client's drinking status ('Ex-drinker', 'Non-drinker', 'Within safe drinking limits' or 'Unsafe - needs intervention'); whether a brief intervention, or a counselling session for alcohol use was provided; whether a relapse prevention medication was prescribed (i.e. acamprosate, naltrexone or disulfiram); and if measured, a range of biological markers which may be affected by alcohol consumption (BP, HbA1C and GGT).

(Insert Figure 4 about here)

A spreadsheet will track dates when key elements of support are provided to each service. Staff numbers attending training and participating in teleconferences will be recorded.

Service retention and withdrawal

Regular contact will be made with services during their support phase through the second monthly teleconferences (by KC and often KH), and for the purposes of data extraction (by BH). Effort will be made to address any concerns or suggestions, and to minimise burden of participation on services. However, services may withdraw from the study, for any reason and at any time.

Data storage and confidentiality

De-identified data will be sent to the project team every two months (by email or Cloudstor). Data will be stored securely in a University of Sydney drive only accessible by relevant members of the research team. Identification numbers rather than names are used for services in the data set and in reporting. Data will stored for seven years after the last publication on the study.

Analysis

Qualitative

Two independent analyses of the qualitative data will be conducted: analysis of the memos and of the transcripts. This complementary analytical strategy aims to enhance examination of the raw data in the formation of key concepts [52].

For peer review only - http://bmjopen.bmj.com/site/about/guidelines.xhtml

For peer review only

Interview transcripts and memos will each be imported into the qualitative software, NVivo (Version 11). Memos will be analysed by the lead interviewer (KH). Transcripts from the structured interviews will be analysed by an independent research associate to produce key themes. This involves the interpretation of descriptive data to explain the meanings of the interviewees' responses [53]. Each data set will be coded separately using a process of constant comparison [53].This will involve coding and organising likened data into categories along with a comparison and interpretation of emerging themes across all transcripts and memos [54]. A team of relevant experts will meet to discuss both analyses and find consensus on key themes and sub-themes. These key themes and findings will be described.

Quantitative

Only the routinely collected data on Indigenous Australian clients aged 16+ years who have attended a participating ACCHS in the past 12 months will be included in analysis to assess:

1. The odds of a client being screened using AUDIT-C at least once in any two month period (out of those who attend in that period)
2. The odds of a client being recorded as receiving treatment for unhealthy alcohol use at the ACCHS, including advice/education or counselling, relapse prevention medicines
3. The number of clients identified by ACCHS staff to have unhealthy alcohol use:
    a. via AUDIT-C: using cut-offs recommended for ACCHSs by the Australian Institute of Health and Welfare (3+ for a woman, 4+ for a man) [23]
    b. and/or staff perception of drinking status (e.g. safe, unsafe, non-drinker or ex-drinker)

We will use statistical packages, SPSS for descriptive analyses and R for multi-level modelling. Multi-level modelling will be used to account for clustering of observations under clients, who are nested within services. We will assess if clients are more likely to be screened for risky alcohol-use and provided with treatment if they are attending a service receiving support.

Two dummy variables will be constructed to indicate whether or not clients were screened with the AUDIT-C during each 2-month data extraction period, or if they received treatment. These

For peer review only - http://bmjopen.bmj.com/site/about/guidelines.xhtml

variables will be used as outcomes in multi-level logistic regressions. As data is aggregated at bi-monthly intervals, repetitive screenings within those two-monthly periods, will not influence the modelled effect of the intervention. However, to ensure that the intervention is associated with increased odds of appropriate screening, we will create a dummy variable indicating whether a client has been screened in the previous 12 months, and use this variable as the outcome in a multilevel logistic regression.

Characteristics of ACCHSs, such as number of clients, will be compared descriptively between the early- and late-support services. Characteristics that display major imbalance between arms will be considered as adjustment factors in outcome analyses. As data is collected as part of routine clinical activity, missing data is likely to be non-random, as such multiple-imputation will not be performed.

All analyses will be repeated for the maintenance phase of the study to see if study effects attenuate or strengthen over time. The analysis will also be repeated after the late support services have received support, to see if any effect on use of screening or treatment can be replicated

If a significant effect of the support on alcohol care outcomes is demonstrated, a secondary analysis will examine the elements of support which were most effective, and whether the effect of support changed over time during a support phase. Potential change in client health indicators over time (AUDIT-C scores, GGT, HbA1C, BP) will be examined in relation to support provision.

Ethical approvals

Ethical approval has been obtained from eight ethics committees: The Aboriginal Health and Medical Research Council of New South Wales (1217/16); Central Australian Human Research Ethics Committee (CA-17-2842); Northern Territory Department of Health and Menzies School of Health Research (2017-2737); Central Queensland Hospital and Health Service (17/QCQ/9); Far North Queensland (17/QCH/45-1143); Aboriginal Health Research Ethics Committee, South

For peer review only - http://bmjopen.bmj.com/site/about/guidelines.xhtml

Australia (04-16-694); St Vincent's Hospital (Melbourne) Human Research Ethics Committee (LRR 036/17); and Western Australian Aboriginal Health Ethics Committee (779).

As there are minimal foreseeable risks in this study, there will not be an independent committee monitoring trial conduct, data or adverse events. Feedback from services will be provided to investigators and adaptations made as necessary.

Patient and public involvement in the research

This trial was suggested by the Aboriginal Health Council SA, the peak organisation for South Australian ACCHSs. Service participation in the trial must be approved by the Board of each local ACCHS. The Board is recognised as representing patients and community in such decisions. As described above, service staff will be consulted throughout their active support phase on how project support can best be tailored. Results will be fed back bimonthly to services in an accessible manner for all staff and board members, and services will have opportunity to provide comment on draft outcome reports before publication.

Dissemination of findings

After feedback to participating ACCHSs, results will be disseminated via peer-reviewed publications and conference presentations. Publications will be led by study investigators, research students or staff supported by the investigators. The resources developed through this trial will also be made freely available electronically at the end of the study period to support other health services.

Data sharing

It is not possible to make the dataset publicly available because of ethical constraints. Alcohol is a sensitive issue, and the data belongs jointly to the 22 services who take part. For the main outcome report, statistical code used to analyse data will be made publicly available.

For peer review only - http://bmjopen.bmj.com/site/about/guidelines.xhtml

For peer review only

Authorship policy

All grant holders, and those involved in study conception and design, will co-author the main outcome piece. Further articles based on sub-analyses or secondary research questions will be authored by those directly involved in those questions. Acknowledgement will be given to individuals involved in design of the main paper.

Protocol amendments

The trial's registration data are shown at Table 2 (ACTRN12618001892202). Any necessary protocol amendments will be agreed on by study investigators, and where appropriate, participating services. Then they will be registered with the Australian and New Zealand Clinical Trials Registry.

For peer review only

For peer review only - http://bmjopen.bmj.com/site/about/guidelines.xhtml

Table 2. WHO trial registration data

Data category	Information
Primary registry and trial identification number	Australia New Zealand Clinical Trials Registry: ACTRN12618001892202
Date of registration in primary registry	2018-11-21
Secondary identifying numbers	APP1105339
Sources of monetary or material support	National Health and Medical Research Council (NHMRC; Australia)
Primary sponsor	The University of Sydney
Secondary sponsor	Royal Prince Alfred Hospital
Contact for public and scientific queries	Kate Conigrave, MBBS, FAChAM, FAFPHM, PhD kate.conigrave@sydney.edu.au
Public title	Supporting Aboriginal Community Controlled Health Services to deliver alcohol care: a cluster randomised controlled trial
Scientific title	Increasing uptake of evidence-based management of unhealthy alcohol use in Aboriginal primary health care services: a cluster randomised controlled trial
Counties of recruitment	Australia
Health condition(s) or problem(s) studied	Hazardous alcohol use; Alcohol use disorders
Interventions	Health service support (including training, sharing learning between services); regular data feedback

For peer review only - http://bmjopen.bmj.com/site/about/guidelines.xhtml

Data category	Information
Key inclusion or exclusion criteria	Health services: 1. are Aboriginal Community Controlled Health services 2. deliver care to at least 1000 unique clients annually 3. use 'Communicare' as their practice software Data from clients 16 years and older are eligible for extraction
Study type	Interventional; Allocation: randomised; Primary purpose: prevention; Cluster randomised trial
Date of first enrolment	28/10/2016
Target sample size	22 services
Recruitment status	Complete
Primary outcomes	AUDIT-C screening rate
Key secondary outcomes	Brief intervention for alcohol rate

For peer review only

For peer review only - http://bmjopen.bmj.com/site/about/guidelines.xhtml

DISCUSSION

To our knowledge, this is the first large-scale randomised trial testing whether external support can enhance uptake of evidence-based alcohol care in services for Indigenous peoples. This study will add knowledge in the field of screening, early intervention and treatment for Indigenous peoples of Australia and inform future research and policy development. The findings are also likely to be relevant for Indigenous peoples internationally who have similar experiences of colonisation and inter-generational trauma. The research will also have relevance for non-Indigenous health services.

A strength of this study is its feasibility across a large number of services due to the use of routinely collected data. However, it is not within the project's resources to assess the quality of data recording. For example, it is likely that some brief discussions of alcohol use will go unrecorded, or will be entered as free text (which is not readily extractable), rather than in the specified fields ('clinical items'). Also, some forms of alcohol risk are not reflected in the AUDIT-C, for example, drinking while pregnant. While we examine health indicators as secondary outcomes (AUDIT-C score, BP, HbA1C, GGT), these may not be sensitive or specific enough to be allow a confident assessment of any reduction in alcohol-related risk or improvement in client health. AUDIT-C scores may in fact become higher with improved quality of screening. In future, data linkage studies (e.g. examining hospital presentations) could allow more definitive assessment of health benefits of a model of service-wide support.

The model of support is designed to be culturally appropriate and collaborative, and tailored to individual service needs. The model is based on the use of champions within services, and feedback of routinely collected data, and so has potential to be sustainable, allowing for continuing quality improvement. The model also has potential to be scaled up for longer-term support of ACCHSs Australia-wide. The need for tailoring and flexibility in support elements provides challenges for analysis. However, this approach is ethically and practically necessary for work in partnership with Indigenous services. This type of local tailoring also has value for non-Indigenous clinical services in culturally and geographically diverse regions.

For peer review only - http://bmjopen.bmj.com/site/about/guidelines.xhtml

Acknowledgements

This work was supported by the NHMRC funding through a Project Grant (#1105339); a Centre of Research Excellence in Indigenous Health and Alcohol (#1117198); and a Practitioner Fellowship for K. Conigrave (#1117582). The funding body had no contribution to study design, writing of report, or in the decision to submit this report for publication; nor will they influence the submission of future reports. Thanks to Dr David Scrimgeour and to the Aboriginal Health Council of South Australia for suggesting this study. Also to Professor Sandra Eades, Professor Robert Sanson-Fisher and Mr Paul Ishiguchi for advice on study design and management.

Authors' contributions

KH refined the protocol for cultural appropriateness, drafted the protocol report; reviewed literature, recruited services, and will play a key role in training, qualitative data collection and qualitative analysis. KC, PH, NH, RI, KL conceived the study design. SW, NH assisted with ensuring design was culturally appropriate; KC,PH,RI, provide clinical expertise; BH and DJ provided guidance on Communicare™ use, data extraction and ACCHS support; BH assisted with service recruitment leads data extraction, and facilitates service liaison. TD developed the analysis plan, including power analyses, and provides ongoing advice. JC is responsible for merging and curating the data, and conducting t_1 quantitative analysis. KC, PH, RI, DG, TD, KL, SW, DJ are grant holders. All grant holders are on the project steering committee; all authors contributed to the development of the study protocol and approved this manuscript.

Declaration of interests

The principal investigators have no financial or other competing interests.

Trial Sponsor

The University of Sydney, NSW 2006, Australia and Sydney Local Health District

For peer review only - http://bmjopen.bmj.com/site/about/guidelines.xhtml

Word Count: 4605 (excluding tables, and acknowledgements onwards)

For peer review only

For peer review only - http://bmjopen.bmj.com/site/about/guidelines.xhtml

References

1. GBD 2016 Risk Factors Collaborators, *Global, regional, and national comparative risk assessment of 84 behavioural, environmental and occupational, and metabolic risks or clusters of risks, 1990–2016: a systematic analysis for the Global Burden of Disease Study 2016.* Global Health Metrics, 2016. **Vol 390 September 16, 2017**.
2. Kirmayer, L.J., G.M. Brass, and C.L. Tait, *The mental health of Aboriginal peoples: transformations of identity and community.* Canadian Journal of Psychiatry, 2000. **45**(7): p. 607-16.
3. Australian Bureau of Statistics/Australian Institute of Health and Welfare, *The health and welfare of Australia's Aboriginal and Torres Strait Islander peoples*. 1999, ABS: Canberra.
4. Australian Institute of Health and Welfare, *Substance use among Aboriginal and Torres Strait Islander people*. 2011, Canberra: Australian Institute of Health and Welfare.
5. Australian Institute of Health and Welfare, *Aboriginal and Torres Strait Islander health performance framework, 2008 report: detailed analyses*. Cat. no. IHW 22. 2008, Canberra: AIHW.
6. Eckermann, A.-K., T. Dowd, and E. Chong, *Binan Goonj: bridging cultures in Aboriginal health*. 2010: Elsevier Australia.
7. World Health Organisation, *ICD-11 for mortality and morbidity statistics (ICD-11 MMS)* June 18 2018 ed. 2018, Geneva: World Health Organisation.
8. Glass, J.E., et al., *Rethinking alcohol interventions in health care: a thematic meeting of the International Network on Brief Interventions for Alcohol & Other Drugs (INEBRIA)*. 2017, BioMed Central.
9. Kaner, E.F., et al., *Effectiveness of brief alcohol interventions in primary care populations.* Cochrane database of systematic reviews, 2018(2).
10. Haber, P., et al., *Guidelines for the treatment of alcohol problems*. 2009, Canberra: Australian Government Department of Health and Ageing.
11. Chapman, C., et al., *Delay to first treatment contact for alcohol use disorder.* Drug and alcohol dependence, 2015. **147**: p. 116-121.
12. Solberg, L.I., M.V. Maciosek, and N.M. Edwards, *Primary care intervention to reduce alcohol misuse: ranking its health impact and cost effectiveness.* American Journal of Preventive Medicine, 2008. **34**(2): p. 143-152.
13. Clifford, A., A. Shakeshaft, and C. Deans, *How and when health-care practitioners in Aboriginal Community Controlled Health Services deliver alcohol screening and brief intervention, and why they don't: A qualitative study.* Drug and Alcohol Review, 2012. **31**(1): p. 13-19.
14. National Health and Medical Research Council, *Australian guidelines to reduce health risks from drinking alcohol*. 2009, Canberra, Australia: Commonwealth of Australia.
15. Reid, A.L.A., et al., *General practitioners' detection of patients with high alcohol intake.* British Medical Journal, 1986. **293**: p. 735-737.
16. d'Abbs, P., et al., *The Grog Mob: lessons from an evaluation of a multi-disciplinary alcohol intervention for Aboriginal clients.* Australian and New Zealand Journal of Public Health, 2013. **37**(5): p. 450-456.
17. Morley, K.C., et al., *National trends in alcohol pharmacotherapy: findings from an Australian claims database.* Drug and alcohol dependence, 2016. **166**: p. 254-257.
18. Ornstein, S.M., et al., *Integration and sustainability of alcohol screening, brief intervention, and pharmacotherapy in primary care settings.* Journal of Studies on Alcohol & Drugs, 2013. **74**(4): p. 598-604.
19. Harris, A.H.S., et al., *Effects of a multifaceted implementation intervention to increase utilization of pharmacological treatments for alcohol use disorders in the US Veterans Health Administration.* Journal of Substance Abuse Treatment, 2017. **82**: p. 107-112.

For peer review only - http://bmjopen.bmj.com/site/about/guidelines.xhtml

20. Campbell, M., J. Hunt, and AHMRC, *Aboriginal communities improving Aboriginal health: An evidence review on the contribution of Aboriginal Community controlled Health Services to improving Aboriginal health*. 2015, Sydney: Aboriginal Health and Medical Research Council, NSW.
21. Clifford, A., A. Shakeshaft, and C. Deans, *Training and tailored outreach support to improve alcohol screening and brief intervention in Aboriginal Community Controlled Health Services.* Drug and Alcohol Review, 2013. **32**(1): p. 72-79.
22. Australian Institute of Health and Welfare, *National Key Performance Indicators for Aboriginal and Torres Strait Islander primary health care: results from June 2016*, in *National key performance indicators for Aboriginal and Torres Strait Islander primary health care series no. 4*. 2017: Canberra.
23. Australian Institute of Health and Welfare, *AIHW National Key Performance Indicators Database User guide reporting period ending 30 June 2017*. 2017.
24. Bradley, K., et al., *AUDIT-C as a brief screen for alcohol misuse in primary care.* Alcoholism: Clinical and Experimental Research, 2007. **31**: p. 1208-17.
25. Babor, T.F., et al., *AUDIT. The Alcohol Use Disorders Identification Test - guidelines for use in primary care*. Second ed. WHO/MSD/MSB/01.6a. 2001, Geneva, Switzerland: World Health Organization, Department of Mental Health and Substance Dependence. 38.
26. Islam, M.M., et al., *Standardised alcohol screening in primary health care services targeting Aboriginal and Torres Strait Islander peoples in Australia.* Addiction Science & Clinical Practice, 2018. **13:5**.
27. Gray, D., et al., *Barriers and enablers to the provision of alcohol treatment among Aboriginal Australians: A thematic review of five research projects.* Drug and Alcohol Review, 2014.
28. Calabria, B., et al., *Identifying Aboriginal-specific AUDIT-C and AUDIT-3 cutoff scores for at-risk, high-risk, and likely dependent drinkers using measures of agreement with the 10-item Alcohol Use Disorders Identification Test.* Addiction Science & Clinical Practice, 2014. **9**(1): p. 17.
29. Keurhorst, M., et al., *Implementation strategies to enhance management of heavy alcohol consumption in primary health care: a meta‐analysis.* Addiction, 2015. **110**(12): p. 1877-1900.
30. Si, D., et al., *Delivery of preventive health services to Indigenous adults: response to a systems-oriented primary care quality improvement intervention.* Medical Journal of Australia, 2007. **187**(8): p. 453-7.
31. Department of Health and Ageing, *Alcohol Treatment Guidelines for Indigenous Australians*. 2007, Commonwealth of Australia: Canberra.
32. National Health and Medical Research Council, *The NHMRC Road Map II: A strategic framework for improving the health of Aboriginal and Torres Strait Isalnder people through research.* 2010.
33. Conigrave, K.M., et al., *Traditional markers of excessive alcohol use [invited review].* Addiction, 2003. **98**(Suppl. 2): p. 31-43.
34. Vancampfort, D., et al., *The prevalence of metabolic syndrome in alcohol use disorders: a systematic review and meta-analysis.* Alcohol and alcoholism, 2016. **51**(5): p. 515-521.
35. Australian Bureau of Statistics, *1216.0 - Australian Standard Geographical Classification (ASGC) - 2006* 2006.
36. Hintze, J., *PASS 12*. 2013, NCSS, LLC: Kaysville, Utah, USA.
37. Australian Bureau of Statistics, *Census of population and housing: characteristics of Aboriginal and Torres Strait Islander Australians, 2011*. 2076.0 2012, Canberra: ABS.
38. Panaretto, K., et al., *Evaluating performance of and organisational capacity to deliver brief interventions in Aboriginal and Torres Strait Islander medical services.* Australian and New Zealand journal of public health, 2010. **34**(1): p. 38-44.

For peer review only - http://bmjopen.bmj.com/site/about/guidelines.xhtml

39. Chikritzhs, T. and M. Brady, *Fact or fiction? ?A critique of the National Aboriginal and Torres Strait Islander Social Survey 2002.* Drug and Alcohol Review, 2006. **25**(3): p. 277-287.
40. Britt, H., et al., *General practice in Australia, health priorities and policy 1998 to 2008*. General practice series no. 24; Cat. no. GEP 24. 2009, Canberra: AIHW.
41. Sanatinia, R., et al., *Brief intervention for alcohol misuse in people attending sexual health clinics: study protocol for a randomized controlled trial.* Trials, 2012. **13**(1): p. 1-8.
42. Coulton, S., et al., *Screening and brief interventions for hazardous alcohol use in accident and emergency departments: a randomised controlled trial protocol.* BMC health services research, 2009. **9**(1): p. 114.
43. Paul, C., et al., *Achieving Type 2 diabetes action and collaborative change in Aboriginal community controlled health services: outcomes of the ADACC cluster randomised trial [abstract].* International Journal of Behavioral Medicine, 2016. **23**: p. S142.
44. Carey M, B.H., Sanson-Fisher R, *The cycle of change: implementing best-evidence clinical practice.* International Journal for Quality in Health Care, 2009. **21**(1): p. 37-43.
45. Kowalyszyn, M. and A. Kelly, *Family functioning, alcohol expectancies and alcohol-related problems in a remote Aboriginal Australian community: a preliminary psychometric validation study.* Drug and Alcohol Review, 2003. **22**(1): p. 53-59.
46. Lee, K., et al., *Handbook for Aboriginal Alcohol and Drug Work*. 2012, Sydney, NSW: University of Sydney.
47. Haber, P., et al., *Quick reference guide to the treatment of alcohol problems: companion document to the guidelines for the treatment of alcohol problems*. 2009, Prepared for the Australian Government Commonwealth Department of Health and Ageing: Canberra. p. 9.
48. Hirsch, A., et al., *Every door closed: Barriers facing parents with criminal records*. 2002, Washington: Community Legal Services, Inc.
49. Ministerial Council on Drug Strategy, *National Drug Strategy Aboriginal and Torres Strait Islander Peoples Complementary Action Plan 2003-2009 (Background Paper)*. 2006, Commonwealth of Australia: Canberra.
50. Sibthorpe BM, et al., *The demise of a planned randomised controlled trial in an urban Aboriginal medical service.* Medical Journal of Australia, 2002. **176**(6): p. 248-9.
51. NSW Health Department, *A Framework for Building Capacity to Improve Health.* 2001.
52. Melanie Birks, Y.C., Karen Francis, *Memoing in qualitative research. Probing data and processes.* Journal of Research in Nursing, 2008. **13**(1): p. 68–75.
53. Morse, J.M. and P.A. Field, *Qualitative research methods for health professionals.* 1995. **2nd edition**: p. 254
54. Glaser, B. and A. Strauss, *The discovery of grounded theory: strategies for qualitative research.* Chicago: Aldine Publishing Company, 1967.

For peer review only

For peer review only - http://bmjopen.bmj.com/site/about/guidelines.xhtml

Figure Legends

Figure 1. Project principles – embedding mainstream evidence into a cultural care approach

Figure 2. Flow diagram for the trial

Figure 3. Three of the elements used to feedback alcohol screening data to ACCHSs

Figure 4: Project Timeline: SPIRIT schedule of enrolment, interventions, and assessments for the trial

For peer review only

For peer review only - http://bmjopen.bmj.com/site/about/guidelines.xhtml

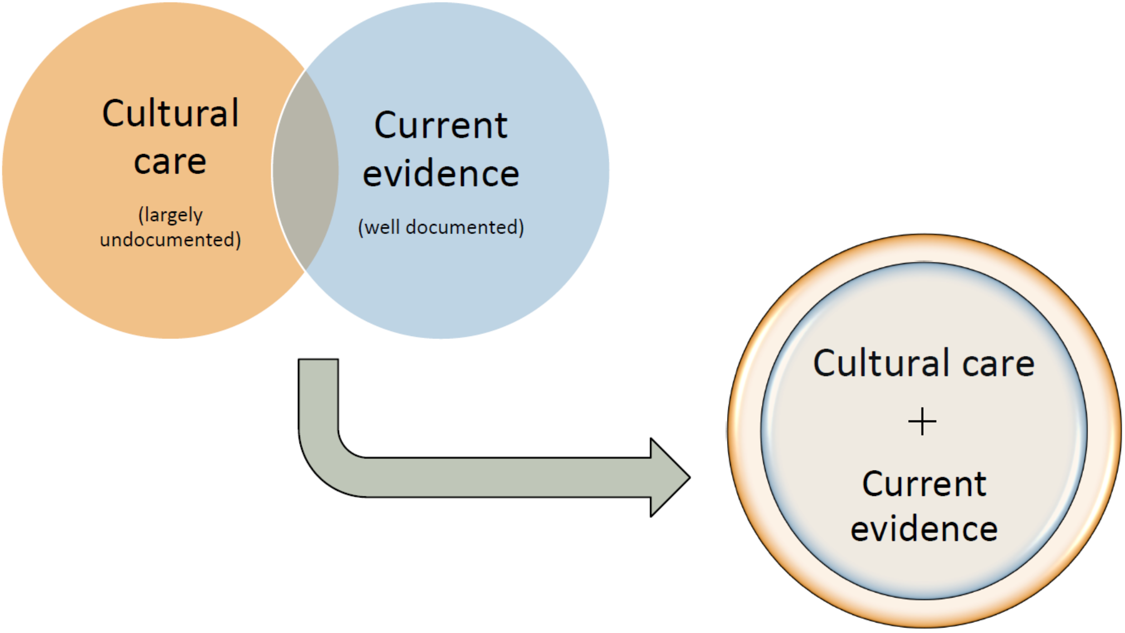
Cultural care
(largely undocumented)
Current evidence
(well documented)
Cultural care
+
Current evidence

review only

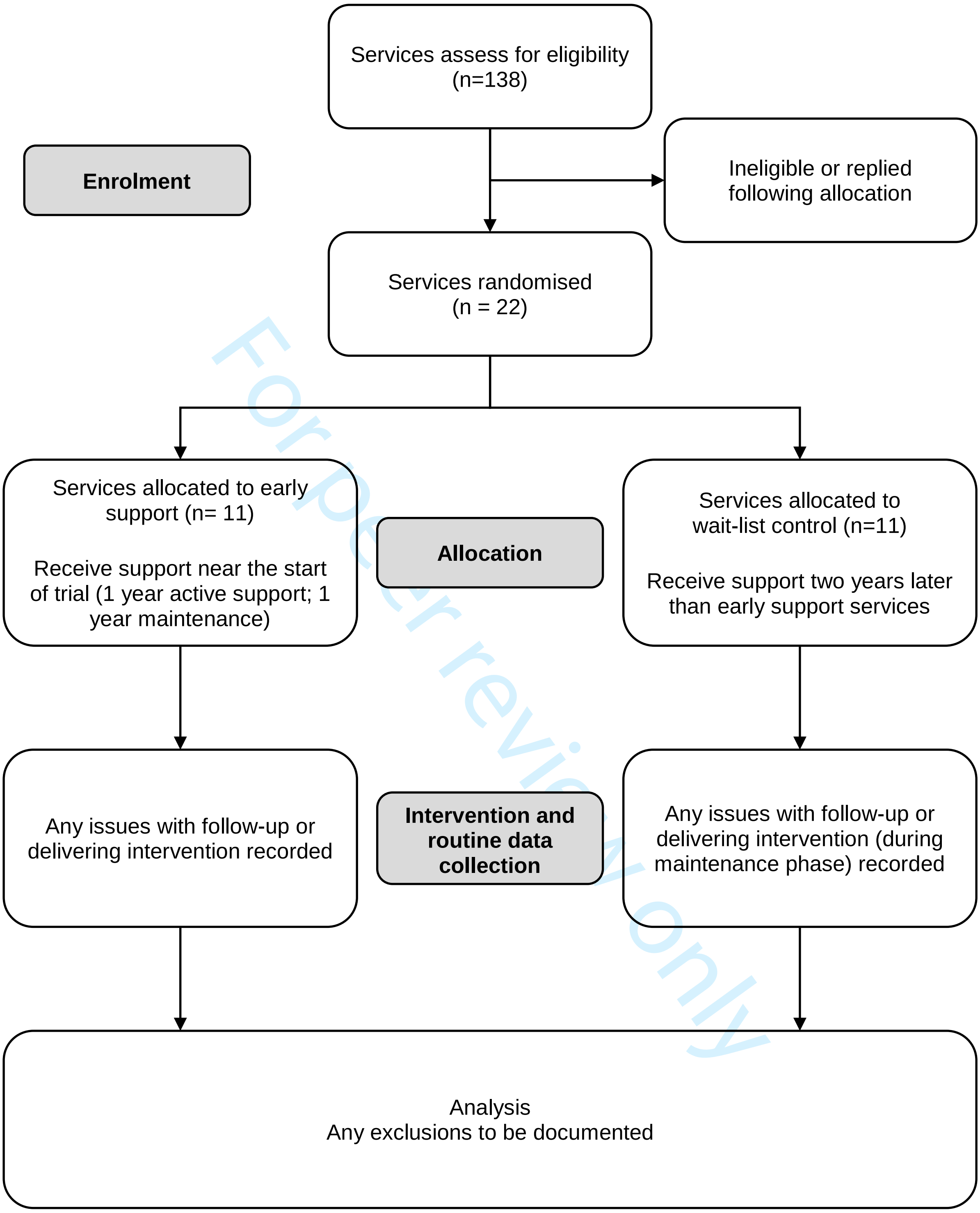

For peer review only

1. Are you meeting your AUDIT-C screening goal?

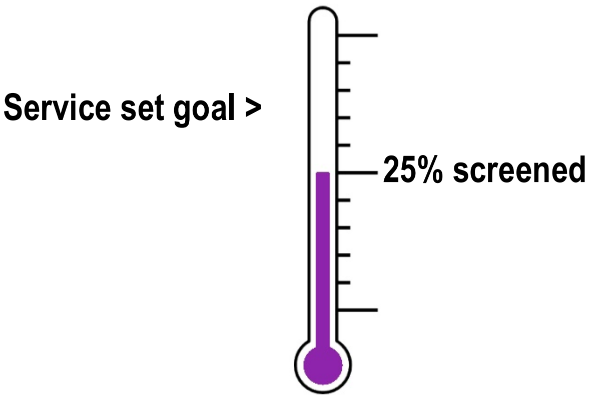

2. How many clients your service is screening

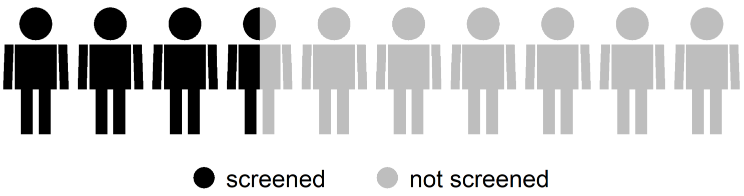

Screening rate over 12 months (out of clients aged 16+ who have attended in the past year)

3. How is your AUDIT-C screening rate going over time?

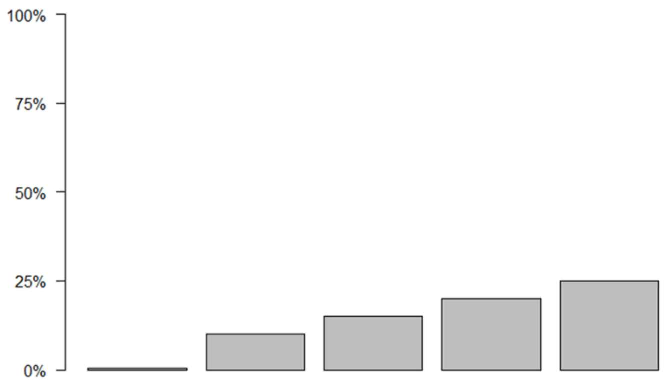

Screening rate for each 2-month period

	Study period						
	Enrolment	Allocation	Post-allocation				
**TIMEPOINT**	***t_{-1} (Feb 16) Oct 2016 to Jun 2017***	**16/06/17**	***t_0 29/8/17 Year 1***	***t_1 28/8/18 Year 2***	***t_2 14/8/19 Year 3***	***t_3 13/8/20 Year 4***	***Close out 28/4/21***
**ENROLMENT:**							
Eligibility screen	X						
Informed consent	X						
Allocation		X					
**INTERVENTIONS:**							
Early support services			α (active)	β (maintenance)			
Late support services (wait-list control)					α (active)	β (maintenance)	
**ASSESSMENTS:**							
**Baseline/outcome variables** Retrospectively collected for 2-monthly periods† from Feb 2016 to Feb 2021: AUDIT-C screening rate; provision of brief interventions‡, counselling; prescriptions for acamprosate, naltrexone, disulfiram; AUDIT-C results; systolic BP; GGT; HbA1c§	X	X	X	X	X	X	X

*Data will be retrospectively extracted for the 12-month period before the start of the support (the intervention). All data used in this study are routinely collected clinical data, extracted from the practice software ('Communicare'). † Data will be extracted on the 28th day of every second month. The first census date (t_1) is one year after the start of the intervention; $t_{2\text{-}4}$ are other census points. ‡ Unhealthy alcohol use = hazardous consumption or alcohol use disorders § Blood test results (GGT, HbA1c) are only available when these have been routinely conducted α Active support phase β Maintenance support phase

SPIRIT

STANDARD PROTOCOL ITEMS: RECOMMENDATIONS FOR INTERVENTIONAL TRIALS

SPIRIT 2013 Checklist: Recommended items to address in a clinical trial protocol and related documents*

Section/item	Item No	Description	Addressed on page number
**Administrative information**			
Title	1	Descriptive title identifying the study design, population, interventions, and, if applicable, trial acronym	i
Trial registration	2a	Trial identifier and registry name. If not yet registered, name of intended registry	iv; Table 2 (p13)
	2b	All items from the World Health Organization Trial Registration Data Set	Table 2 (p13)
Protocol version	3	Date and version identifier	iv
Funding	4	Sources and types of financial, material, and other support	15
Roles and responsibilities	5a	Names, affiliations, and roles of protocol contributors	i, 15
	5b	Name and contact information for the trial sponsor	15
	5c	Role of study sponsor and funders, if any, in study design; collection, management, analysis, and interpretation of data; writing of the report; and the decision to submit the report for publication, including whether they will have ultimate authority over any of these activities	15
	5d	Composition, roles, and responsibilities of the coordinating centre, steering committee, endpoint adjudication committee, data management team, and other individuals or groups overseeing the trial, if applicable (see Item 21a for data monitoring committee)	i, 15

For peer review only

For peer review only - http://bmjopen.bmj.com/site/about/guidelines.xhtml

Introduction

Background and rationale	6a	Description of research question and justification for undertaking the trial, including summary of relevant studies (published and unpublished) examining benefits and harms for each intervention	1 - 3
	6b	Explanation for choice of comparators	2
Objectives	7	Specific objectives or hypotheses	3
Trial design	8	Description of trial design including type of trial (eg, parallel group, crossover, factorial, single group), allocation ratio, and framework (eg, superiority, equivalence, noninferiority, exploratory)	3

Methods: Participants, interventions, and outcomes

Study setting	9	Description of study settings (eg, community clinic, academic hospital) and list of countries where data will be collected. Reference to where list of study sites can be obtained	3
Eligibility criteria	10	Inclusion and exclusion criteria for participants. If applicable, eligibility criteria for study centres and individuals who will perform the interventions (eg, surgeons, psychotherapists)	4
Interventions	11a	Interventions for each group with sufficient detail to allow replication, including how and when they will be administered	6 – 9; Figure 2 (p3)
	11b	Criteria for discontinuing or modifying allocated interventions for a given trial participant (eg, drug dose change in response to harms, participant request, or improving/worsening disease)	12
	11c	Strategies to improve adherence to intervention protocols, and any procedures for monitoring adherence (eg, drug tablet return, laboratory tests)	10
	11d	Relevant concomitant care and interventions that are permitted or prohibited during the trial	N/A
Outcomes	12	Primary, secondary, and other outcomes, including the specific measurement variable (eg, systolic blood pressure), analysis metric (eg, change from baseline, final value, time to event), method of aggregation (eg, median, proportion), and time point for each outcome. Explanation of the clinical relevance of chosen efficacy and harm outcomes is strongly recommended	4
Participant timeline	13	Time schedule of enrolment, interventions (including any run-ins and washouts), assessments, and visits for participants. A schematic diagram is highly recommended (see Figure)	6, Figures 2 (p3)

For peer review only - http://bmjopen.bmj.com/site/about/guidelines.xhtml

Sample size	14	Estimated number of participants needed to achieve study objectives and how it was determined, including clinical and statistical assumptions supporting any sample size calculations	5
Recruitment	15	Strategies for achieving adequate participant enrolment to reach target sample size	4
**Methods: Assignment of interventions (for controlled trials)**			
Allocation:			
Sequence generation	16a	Method of generating the allocation sequence (eg, computer-generated random numbers), and list of any factors for stratification. To reduce predictability of a random sequence, details of any planned restriction (eg, blocking) should be provided in a separate document that is unavailable to those who enrol participants or assign interventions	5
Allocation concealment mechanism	16b	Mechanism of implementing the allocation sequence (eg, central telephone; sequentially numbered, opaque, sealed envelopes), describing any steps to conceal the sequence until interventions are assigned	5
Implementation	16c	Who will generate the allocation sequence, who will enrol participants, and who will assign participants to interventions	4-5
Blinding (masking)	17a	Who will be blinded after assignment to interventions (eg, trial participants, care providers, outcome assessors, data analysts), and how	6
	17b	If blinded, circumstances under which unblinding is permissible, and procedure for revealing a participant's allocated intervention during the trial	N/A
**Methods: Data collection, management, and analysis**			
Data collection methods	18a	Plans for assessment and collection of outcome, baseline, and other trial data, including any related processes to promote data quality (eg, duplicate measurements, training of assessors) and a description of study instruments (eg, questionnaires, laboratory tests) along with their reliability and validity, if known. Reference to where data collection forms can be found, if not in the protocol	9-10
	18b	Plans to promote participant retention and complete follow-up, including list of any outcome data to be collected for participants who discontinue or deviate from intervention protocols	10

For peer review only - http://bmjopen.bmj.com/site/about/guidelines.xhtml

Data management	19	Plans for data entry, coding, security, and storage, including any related processes to promote data quality (eg, double data entry; range checks for data values). Reference to where details of data management procedures can be found, if not in the protocol	10
Statistical methods	20a	Statistical methods for analysing primary and secondary outcomes. Reference to where other details of the statistical analysis plan can be found, if not in the protocol	11-12
	20b	Methods for any additional analyses (eg, subgroup and adjusted analyses)	11-12
	20c	Definition of analysis population relating to protocol non-adherence (eg, as randomised analysis), and any statistical methods to handle missing data (eg, multiple imputation).	12
**Methods: Monitoring**			
Data monitoring	21a	Composition of data monitoring committee (DMC); summary of its role and reporting structure; statement of whether it is independent from the sponsor and competing interests; and reference to where further details about its charter can be found, if not in the protocol. Alternatively, an explanation of why a DMC is not needed **It's mainly used where treatment can cause harms, here minimal risk**	12
	21b	Description of any interim analyses and stopping guidelines, including who will have access to these interim results and make the final decision to terminate the trial	N/A
Harms	22	Plans for collecting, assessing, reporting, and managing solicited and spontaneously reported adverse events and other unintended effects of trial interventions or trial conduct	10
Auditing	23	Frequency and procedures for auditing trial conduct, if any, and whether the process will be independent from investigators and the sponsor	12
**Ethics and dissemination**			
Research ethics approval	24	Plans for seeking research ethics committee/institutional review board (REC/IRB) approval	12
Protocol amendments	25	Plans for communicating important protocol modifications (eg, changes to eligibility criteria, outcomes, analyses) to relevant parties (eg, investigators, REC/IRBs, trial participants, trial registries, journals, regulators)	14

For peer review only - http://bmjopen.bmj.com/site/about/guidelines.xhtml

Consent or assent	26a	Who will obtain informed consent or assent from potential trial participants or authorised surrogates, and how (see Item 32)	4-5
	26b	Additional consent provisions for collection and use of participant data and biological specimens in ancillary studies, if applicable	5
Confidentiality	27	How personal information about potential and enrolled participants will be collected, shared, and maintained in order to protect confidentiality before, during, and after the trial. **do we need to mention data destroyed 7 years after last publication)?	10
Declaration of interests	28	Financial and other competing interests for principal investigators for the overall trial and each study site	15
Access to data	29	Statement of who will have access to the final trial dataset, and disclosure of contractual agreements that limit such access for investigators	10
Ancillary and post-trial care	30	Provisions, if any, for ancillary and post-trial care, and for compensation to those who suffer harm from trial participation	N/A
Dissemination policy	31a	Plans for investigators and sponsor to communicate trial results to participants, healthcare professionals, the public, and other relevant groups (eg, via publication, reporting in results databases, or other data sharing arrangements), including any publication restrictions	13
	31b	Authorship eligibility guidelines and any intended use of professional writers	13
	31c	Plans, if any, for granting public access to the full protocol, participant-level dataset, and statistical code	13
**Appendices**			
Informed consent materials	32	Model consent form and other related documentation given to participants and authorised surrogates	Appendices 1-4
Biological specimens	33	Plans for collection, laboratory evaluation, and storage of biological specimens for genetic or molecular analysis in the current trial and for future use in ancillary studies, if applicable	NA

*It is strongly recommended that this checklist be read in conjunction with the SPIRIT 2013 Explanation & Elaboration for important clarification on the items. Amendments to the protocol should be tracked and dated. The SPIRIT checklist is copyrighted by the SPIRIT Group under the Creative Commons "Attribution-NonCommercial-NoDerivs 3.0 Unported" license.

For peer review only - http://bmjopen.bmj.com/site/about/guidelines.xhtml
